# A scoping review of outdoor food marketing: exposure, power and impacts on eating behaviour and health

**DOI:** 10.1186/s12889-022-13784-8

**Published:** 2022-07-27

**Authors:** Amy Finlay, Eric Robinson, Andrew Jones, Michelle Maden, Caroline Cerny, Magdalena Muc, Rebecca Evans, Harriet Makin, Emma Boyland

**Affiliations:** 1 Department of Psychology, University of Liverpool, Liverpool, L69 7ZA UK; 2 Liverpool Reviews and Implementation Group, University of Liverpool, Liverpool, L69 3GB UK; 3 Obesity Health Alliance, Liverpool, UK

**Keywords:** Outdoor, Advertising, Food, HFSS

## Abstract

**Background:**

There is convincing evidence that unhealthy food marketing is extensive on television and in digital media, uses powerful persuasive techniques, and impacts dietary choices and consumption, particularly in children. It is less clear whether this is also the case for outdoor food marketing. This review (i) identifies common criteria used to define outdoor food marketing, (ii) summarises research methodologies used, (iii) identifies available evidence on the exposure, power (i.e. persuasive creative strategies within marketing) and impact of outdoor food marketing on behaviour and health and (iv) identifies knowledge gaps and directions for future research.

**Methods:**

A systematic search was conducted of Medline (Ovid), Scopus, Science Direct, Proquest, PsycINFO, CINAHL, PubMed, the Cochrane Database of Systematic Reviews, the Cochrane Central Register of Controlled Trials and a number of grey literature sources. Titles and abstracts were screened by one researcher. Relevant full texts were independently checked by two researchers against eligibility criteria.

**Results:**

Fifty-three studies were conducted across twenty-one countries. The majority of studies (*n* = 39) were conducted in high-income countries. All measured the extent of exposure to outdoor food marketing, twelve also assessed power and three measured impact on behavioural or health outcomes. Criteria used to define outdoor food marketing and methodologies adopted were highly variable across studies. Almost a quarter of advertisements across all studies were for food (mean of 22.1%) and the majority of advertised foods were unhealthy (mean of 63%). The evidence on differences in exposure by SES is heterogenous, which makes it difficult to draw conclusions, however the research suggests that ethnic minority groups have a higher likelihood of exposure to food marketing outdoors. The most frequent persuasive creative strategies were premium offers and use of characters. There was limited evidence on the relationship between exposure to outdoor food marketing and eating behaviour or health outcomes.

**Conclusions:**

This review highlights the extent of unhealthy outdoor food marketing globally and the powerful methods used within this marketing. There is a need for consistency in defining and measuring outdoor food marketing to enable comparison across time and place. Future research should attempt to measure direct impacts on behaviour and health.

**Supplementary Information:**

The online version contains supplementary material available at 10.1186/s12889-022-13784-8.

## Background

Advertising of foods and non-alcoholic beverages, (hereafter food advertising), particularly for items high in fat, salt and/or sugar (HFSS), has been identified as a factor contributing to obesity and associated non-communicable diseases globally [1]. People from more deprived backgrounds or ethnic minority groups are disproportionately targeted and exposed to greater food marketing across a range of platforms [2], and this may contribute to social gradients in obesity and associated health inequalities [3]. Marketing is defined by the American Marketing Association (AMA) as “the activity, set of institutions, and processes for creating, communicating, delivering, and exchanging offerings that have value for customers, clients, partners, and society at large” [4], and advertising is a key aspect of marketing, which seeks to “inform and/or persuade members of a particular target market or audience regarding their products, services, organizations or ideas” [5]. The World Health Organization (WHO) assert that the impact that food marketing has on consumer behaviour is dependent on both ‘exposure’ and ‘power’ [6]. Exposure is the frequency and reach of the marketing messages and power is the creative content and strategies used, both of which determine the effectiveness of marketing [6]. Hierarchy of effects models of food marketing consider that the pathways for these effects are likely to be complex [7], with evidence demonstrating that food marketing impacts food purchasing [8], purchase requests [9], consumption [10, 11] and obesity prevalence [12].

Evidence suggests that children are likely to be more vulnerable to marketing messages than adults [13–15]. Furthermore, it has been proposed that the scepticism towards advertising that is developed in adolescence does not equate to protection against its effects [16], leaving both young children and older adolescents vulnerable to the effects of food marketing [17]. For this reason, policies enacted generally aim to decrease the exposure or power of food marketing to children, and so this is where much of the research is focused. Despite this, it is apparent that adults are similarly affected by food marketing [18], and therefore also likely to benefit from restrictions [19].

In 2010, WHO called on countries to limit the marketing of unhealthy foods, specifically to children [6]. Various policies have since attempted to enforce restrictions on HFSS advertisements [20], however, restrictions outdoors remain scarce [21] and implementation and observation of such restrictions has been found to be inconsistent and problematic [22].

Previous reviews have collated the evidence on the exposure, power and impact of food advertising on television [23–25], advergames [26, 27], sports sponsorship [28, 29] and food packaging [30, 31] and in some cases across a range of mediums [2, 32]. An existing scoping review [33] documents the policies in place globally to target outdoor food marketing, and the facilitators and barriers involved in implementing these policies. The lack of effective policies for outdoor food marketing may reflect the comparatively little evidence or synthesis of evidence on outdoor marketing or its potential role in contributing to overweight and obesity, relative to that for other media. Additionally, there are challenges in measuring outdoor marketing exposure compared to television and online [34]. As countries such as the UK and Chile [35] move to strengthen restrictions on unhealthy food marketing via television, digital media and packaging, it is plausible that advertisements will be displaced to other media such as outdoor mediums so that brands can maintain or increase their exposure [36, 37].

Despite being a longstanding and widely used format [38] there is no agreed definition for outdoor food marketing. This may have implications for the comparability of data across study designs, which has been reported as a limitation in previous reviews [11, 39]. Identifying the common criteria used to define outdoor food marketing, alongside considering best practice methodologies for outdoor marketing monitoring and impact research, are important steps to support the generation of robust, comparable evidence to underpin public health policy development.

Given that 98% of people are exposed to outdoor marketing daily [40], it is an efficient form of marketing for brands [41], and is likely successful in influencing purchase decisions through targeting potential shoppers in places the brands are sold [42]. Food marketing through media such as television and advergames have been shown to impact eating and related behaviours such as purchasing [43–46], and the evidence on this marketing and body weight has satisfied the Bradford Hill Criteria [47], which is used to recognise a causal relationship between two variables. However, the impact that outdoor marketing has on eating related outcomes is less clear.

Therefore, this scoping review aims to (i) identify common criteria used to define outdoor food marketing, (ii) summarise research methodologies used, (iii) identify available evidence on the exposure, power (i.e. persuasive creative strategies within marketing) and impact of outdoor food marketing on behaviour and health with consideration of any observed differences by equity characteristics such as socioeconomic position and (iv) identify knowledge gaps and directions for future research.

## Methods

### Approach

Given the broad objectives, a scoping review [48] was conducted and reported in accordance with the Joanna Briggs Institute (JBI) methodology for scoping reviews [49] and the Preferred Reporting Items for Systematic reviews and Meta-Analyses extension for scoping reviews (PRISMA-ScR) [50]. The review was pre-registered on the Open Science Framework (https://osf.io/wezug).

### Search strategy

A detailed search strategy was created by the research team (see supplementary material 1), which included an experienced information specialist (M.Ma), to capture both published and unpublished studies and grey literature. Search terms related to food, outdoor and marketing were developed based on titles and abstracts of key studies (identified from preliminary scoping searches) and index terms used to describe articles. For grey literature sources simple terms “outdoor food marketing” and “outdoor food advertising” were used. Searches were conducted between 21st January and 10th February 2021.

Databases searched for academic literature included Medline (Ovid), Scopus, Science Direct, Proquest, PsycINFO, CINAHL, the Cochrane Database of Systematic Reviews and the Cochrane Central Register of Controlled Trials. An additional supplementary PubMed search was conducted to ensure journals and manuscripts in PubMed Central and the NCBI bookshelf were captured. Grey literature searches were conducted of databases Open Access Theses and Dissertations, OpenGrey, UK Health Forum, WHO and Public Health England. Other searches for grey literature included government websites (GOV.uk), regulatory and industry body websites (World Advertising Research Centre Database, Advertising Standards Authority) and NGO sites (Obesity Health Alliance, Sustain).

### Eligibility criteria

Primary quantitative studies assessing marketing of food and non-alcoholic beverage brands or products encountered outdoors in terms of exposure, power or impact were considered for inclusion. We defined both marketing and advertising as per the AMA definitions [4, 5].

Examples of outdoor marketing included billboards, posters, street furniture and public transport. Exposure was defined as the volume of advertising identified, with consideration of the brands and products promoted. Power of outdoor marketing was defined as the strategies used to promote products (e.g. promotions, characters) [51]. Eligible behavioural impacts of outdoor marketing were food preference, choice, purchase, intended purchase, purchase requests and consumption. Health-related impacts were body weight and prevalence of obesity or non-communicable diseases. Non-behavioural outcomes were ineligible, e.g., brand recall, awareness, or attitudes.

Studies in which outdoor marketing could not be clearly isolated from other marketing forms [46], or food could not be isolated from other marketed products (e.g. alcohol and tobacco) were excluded. Studies of health promotion (e.g., public health campaigns) were ineligible. Qualitative studies and reviews were not eligible for inclusion; however, reference lists of relevant reviews were searched.

### Selection of sources of evidence

**Fig. 1:**
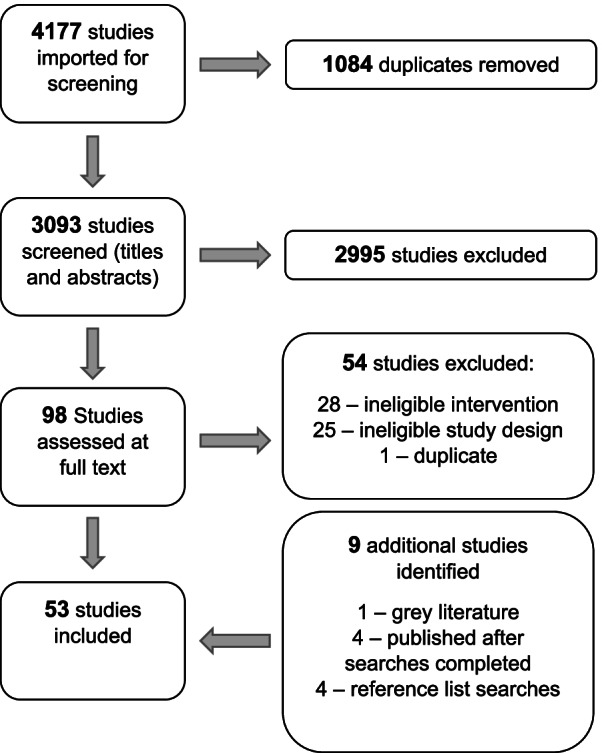
PRISMA flow diagram

The full screening process is shown as a PRISMA flow diagram (Fig. 1). Titles and abstracts were screened by one researcher (AF). Full text review was conducted independently by two researchers from a pool of four (AF, M. Mu, RE & HM). Disagreement was resolved by discussion and where necessary (*n* = 4 articles) a third reviewer (EB) was consulted. Covidence systematic review software was used to organise the screening of studies. Inter-rater reliability for the full-text screening was high, with estimated agreement of 95.7% and a Kappa score of 0.91.

### Data charting

The extraction template was developed and piloted prior to data extraction. For more detail on the information extracted from each article see supplementary material 2. Discrepancies in extraction were resolved by discussion. As the aim was to characterise and map existing literature and not systematically review its quality, as is common in scoping reviews [52] quality assessment (e.g. risk of bias) was not undertaken.

### Synthesis of results

Studies that defined outdoor food marketing were grouped to identify common criteria used in definitions. Methodologies used to measure exposure, power and impact are summarised. Studies were grouped into exposure, power and impact for synthesis, with relevant sub-categories to document findings related to equity characteristics. We deemed foods classed as “non-core”, “discretionary”, “unhealthy”, “less healthy”, “junk”, “HFSS”, “processed”, “ultra-processed”, “occasional”, “do not sell”, “poorest choice for health”, “less healthful”, “ineligible to be advertised”, and “not permitted” as unhealthy.

## Results

### Study selection

After removal of duplicates from an initial 4177 records, 3093 records were screened. Ninety-eight articles were then full-text reviewed. Fifty-four studies were excluded here (supplementary material 3). After grey literature and citation searches, the final number of included studies was 53.

### Characteristics of included studies

All studies (*n* = 53) measured exposure to outdoor food marketing, *n* = 12 also measured power of outdoor food marketing, and *n* = 3 measured impact. *N* = 15 studies provided at least one criterion through which outdoor food marketing was defined, beyond stating the media explored.

Studies were conducted across twenty-one countries, the majority took place in the USA (*n* = 16) [53–68], and other high-income countries (*n* = 23) as categorised by the world bank [69] (Tables 1, 2 and 3).

**Table 1:** Studies measuring exposure and impact of outdoor food marketing

Author(s), year of publication	Study objectives	Study setting	Study Design, participants	Methods	Outdoor Advertising mediums included	Main results relating to outdoor advertising
Fernandez et al. (2019) [70]	To determine the association between food marketing exposure and consumption of confectioneries among pre-school children in Central Jakarta.	Early childhood education centres/ schools in Jakarta, Indonesia	Cross-sectional questionnaire. 240 caregivers of preschool children aged 3–5. (79.2% mothers)	Caregivers interviewed on frequency of intake of 26 confectionaries at home and frequency of food marketing exposure in the past week.	Advertising seen on public transport	35.4% of participants reported exposure to advertising on public transport at least once in the last week. Exposure to food advertising on public transport was associated with consumption of two products - Beng-beng (wafer crisp covered with chocolate; *p* = .006) and Walls (ice cream cup; *p* < .001). No significant association between exposure to food marketing on transport and consumption of the remaining 8 most consumed confectionaries.
Lesser et al. (2013) [53]	To investigate whether individuals living in areas with higher proportions of outdoor food advertising, compared to those in living in areas with lower amounts, have greater odds of obesity and a higher rate of soda consumption.	Los Angeles and Louisiana, USA	Cross-sectional survey and content analysis. A systematic sample of 2589 adults from geographically referenced telephone-listed households from census tracts.	Teams visited each location, following the perimeter then going through each street recording all ads classified as alcohol, tobacco, food and/or restaurants and other. Documented GPS of each ad, format used and frequency of each ad. Telephone interviews collected data on height, weight, and how many 12 oz. sodas were consumed in the last 24 hours.	Posters, flyers, flags, banners, transit shelters or benches, billboards, Storefront ads excluded	69.4% of all areas had any outdoor ads, 44.3% had outdoor F&B ads. On average, 10.4% of ads were related to F&B. LA had significantly more ads per census tract than New Orleans, Louisiana (*p* < .001, 14 v. 6) but a lower percentage of food ads (*p* < 0.05, 6% v. 15%). Participants on average drank 1.3 12 oz. sodas per day, every 10% increase in food ads was associated with a 6% increase in sodas consumed (IRR 1.06, 95%CI 1.03 to 1.0, *p* < .001), and odds of obesity increased by 5% (OR 1.05, 95%CI 1.0 to 1.10, *p* < .03). *PROGRESS*: Low-income areas with a majority Black population were 2.59 times more likely to have any food ads than high-income white areas (95% CI = 1.04–6.48), whilst low-income areas with majority Latino residents were 3.1 times more likely to have any food ads compared to high-income white areas (95% CI = 1.03–9.2). There was no significant relationship between census tract characteristics and % of food ads.
Yau et al. (2021) [71]	To explore sociodemographic differences in exposure to advertising for foods and drinks high in fat, salt and sugar (HFSS) and whether exposure is associated with body mass index (BMI).	London and the North of England	Cross-sectional Survey The main food shopper of 1552 households.	Assessed self-reported exposure (last 7 days) to advertising for HFSS processed foods, sugary drinks, sugary cereals, sweet snacks, fast food and food delivery services in 5 different settings (traditional, digital, recreational, functional, transport). Answers were coded as exposed or not. BMI calculated using self-reported data (available for 81.7% of participants).	Transport (outside/inside buses, tube, tram or train stations, bus stops, taxi and back of bus ticket)	36.4% of participants reported exposure to food advertising across transport networks. There was no significant relationship between exposure on public transport and weight status. *PROGRESS:* No significant differences in food advertising exposure across transport by socioeconomic position. Full time employees were 50% more likely to report advertising exposure on public transport than those not looking for, or unable to work. *Demographic:* 18–34 year olds were 96% more likely to report exposure to advertising on public transport than > 65 s. But odds of reporting exposure were not significantly higher in age groups 35–44, 45–54 and 55–64 than ≥65.

*Abbreviations*: *HFSS* Foods high in fats, salt, or sugar *F&B* Food and beverage, *Ads* Advertisements, *WHO* World Health Organization

**Table 2:** Studies measuring exposure and power of outdoor food marketing

Author(s), year of publication	Study objectives	Study setting	Study Design	Methods	Advertising mediums included	Main results relating to outdoor advertising
Amanzadeh et al. (2015) [72]	To discern the strategies and messages used to promote consumption of highly processed, commercialized products and how these differ between rural and urban areas.	Convenience sample of 100 ads around 6 rural villages and the roads connecting them to Santa Ana (El Salvador), and the road from Santa Ana to San Salvador.	Cross-sectional, observational, visual interpretive	Photographs were taken of 100 fast food, snack food, and beverage ads (including beer) were taken during a one-week period in July 2010. Fifty-three from rural areas and 47 from urban. Ads coded for location (wall, billboard, other), type of product, visual details, placement and context. Inferential meaning and main theme of each ad was identified.	Billboard and wall ads	In 100 unique ads, there were 31 pictures of fast food (0 rural, 31 urban) and 40 pictures of snacks (31 rural, 9 urban) *Power*: “Cheap price, large and fast” and “modern” present in fast food ads. “refreshment”, “sports and nationalism” and “sex and gender roles” seen in SSB ads, “fun and happy feelings” often conveyed in snack food ads Communication strategies identified include: Combination (e.g. refreshment and sports theme in a soda ad), repetition, placement/visibility, personification (e.g. cartoon characters), redefining food and meals (promoting modern lifestyle of fast food and snacking on the run) There were no rural fast food ads due to a lack of establishments. In urban areas, approximately 66% (31/47) of ads identified were for fast food. *Note: additional food data was not extracted due to being combined with alcoholic beverages*
Bragg et al. (2017)a [73]	(1) assess the marketing themes and sugar content of beverages promoted in outdoor ads within a portion of Accra, Ghana and (2) quantify the types of ads that appeared along the Accra-Cape Coast Highway.	A randomly chosen 5 km squared region of Accra, Ghana on two-lane roads (77 ads) and along the Accra-Cape Coast Highway (a 151 km highway; 56 ads)	Cross-sectional, observational content analysis	1) Researchers took photographs of non-alcoholic beverage ads. Ads had to be visible from the street. Items were classified as SSB or non-SSB. Presence of child-directed marketing, cultural relevance, health/fitness references were documented. 2) Researchers tracked the number and type of all beverage ads present on the Accra-Cape Coast Highway	Small sign, large billboard, front-of-store promotional display. Billboards defined as large signs with dimensions greater than 0.61× 0.91 m and front-of-store displays as small signs with dimensions less than 21.59 × 27.94 cm.	1) Researchers captured 77 non-alcoholic beverage ads, from brands including Coca Cola (59.7%) Pepsi (3.9%) and Nestle (22.1%). 72.7% featured SSBs. 64.9% of non-alcoholic beverage ads promoted sodas, the remaining 35.1% promoted energy drinks(*n* = 1), bottled or canned juice (*n* = 3), coffee-based (*n* = 3), milk-based (*n* = 15) or water-based (*n* = 5) beverages. *PROGRESS:* 46.8% of ads used an aspect relevant to Ghanaian culture. *Power:* 46.8% featured a person or a character, 9.1% contained a ‘healthy’ reference. 7.8% contained a word conveying fitness, strength or sport. *Demographic:* 10.4% were child targeted, 5.2% of ads were directly next to something identified as child-oriented (e.g. school) 2) On the Accra-Cape Coast Highway, 60% of ads featuring a beverage logo were associated with Coca cola, 20% with Pepsi, and 20% with Nestle. Where a beverage was shown, 64.3% were for local fruit and vegetable drinks, the second most frequent seen were local SSBs (19.6%).
Bragg et al. (2017)b [54]	1) To quantify the number and type of ads located in a Chinese-American neighbourhood in a large, urban city. 2) Catalogue the targeted marketing themes used in the food/beverage ads.	Chinese-American neighbourhoods in New York City with over 60% of residents identify as Asian American (1366 ads)	Cross-sectional, observational content analysis	Pairs of research assistants photographed F&B ads in 0.6mile^2^ area. Content analysis was used to assess marketing themes (e.g. reference to Asian culture, health, various languages, children) and ads were coded according to F&B type.	Billboards, front of store displays, signs, excluding graffiti	There were 183 food only ads (13.4% of total. There were 113 SSB ads (8.3% of total, 66.9% of beverages). The most frequent products in food only ads were fast food (42.5%, *n* = 89), candy/desserts (24.8%, *n* = 52), snacks (14.8%, *n* = 31), fruit (9%, *n* = 19), restaurants (4.7%, *n* = 10) condiments (2.8%, *n* = 6) and vegetables (0.9%, *n* = 2). *Power/PROGRESS*: 50.3% of food only ads were Asian foods, 43.7% non-Asian-American. 27.5% were Chinese-targeted (e.g. Asian model or flag), 21.6% used Chinese language. 59% of non-alcoholic beverage ads (calculated without alcohol from available data) were Chinese-targeted. 45.8% used Chinese language *Demographic:* 8.2% were child-targeted *Note: additional food data was not extracted due to being combined with alcoholic beverages*
Busse (2018) [74]	To identify: (1) The types of food products that children and adolescents frequently find (2) The persuasive techniques used (3) The extent that eating behaviors are portrayed in advertising?	363 billboards in the top 51 locations with heaviest traffic in Lima, Peru	Cross-sectional, observational, content analysis	Billboards 300 m to the left or right of the reference point of each traffic location were included and photographs taken. Most were static images, but 1/3 digital videos. Researchers coded type of advertised product, and all food ads were coded further according to food type, persuasive appeals- and portrayal of eating behaviour.	Billboards	F&B ads comprised of 14.05% (51 of 363 billboards). Most frequently advertised on billboards were pizza and fast food restaurants (39.22%, *n* = 20) followed by sweets (27.45%, *n* = 14), sugary drinks (9.8%, *n* = 5), supermarkets (7.84%, *n* = 4) and dairy (5.88%, *n* = 3). *Power:* Of ads using persuasive appeals, strategies included flavour, taste, smell, texture (70.59%), competitive/unique (21.57%), new/innovative (21.57%), mood alterations (13.73%), other (13.73%), nutritional content (11.76%), family bonding (11.76%), fun (9.8%), national pride (7.84%), achievement/enablement (7.84%), premium offers (7.84%), health/well-being (5.88%), value for money (3.92).
Dia et al. (2021) [75]	To map the outdoor F&B advertising environment in terms of extent and power, around selected schools in the capital of Uganda, Kampala.	250 m radius around 25 schools in two of five divisions of Kampala, Uganda. (1034 F&B ads).	Cross-sectional, observational, content analysis	Data was collected on the distance of F&B ads from school, size of ad, setting, type and position of ad, single or multiple foods present, brand name, product type, food category, promotional characters/premium offers. Ads were considered unhealthy when at least one food product in the ad was categorised as unhealthy following the Outdoor Advertising protocol and WHO NPM	Billboards, posters, free standing signs, neon stickers, electronic boards banners, bus shelter signs and signs on outdoor furniture, bridge/awning signs and painted buildings, as well as store signage with a product logo and served not just as a store identifier but also as promotional material for a product	1034 F&B ads were identified, 63% (*n* = 654) were for unhealthy foods and non-alcoholic beverages (calculated without alcohol from available data), 7% for healthy foods and 7% for miscellaneous. The most frequently advertised food products were SSBs (51% of food ads including alcohol), high fat and/or sugar flavoured dairy products (5%), condiments, seasonings and recipe additions (5%), bottled water (3%) and chocolate and candy (2%). For 88% of schools, SSBs were the most promoted product, within SSBs, coca cola accounted for 67%. 24 schools had between 0 and 7 healthy ads within 250 m, although one school had 15. 23 schools had between 0 and 7 miscellaneous ads, two schools had 10 and 11. *Power:* Promotional characters were present in 15% of healthy food ads. *PROGRESS:* The number of healthy food ads was significantly higher in urban areas (higher income) than peri-urban (*p* = 0.005), although still infrequent (median of 4 per school). There was no significant difference in the number of healthy food ads around government funded vs private schools. *Note: additional food data was not extracted due to being combined with alcoholic beverages*
Herrera & Pasch (2018) [55]	To determine if the prevalence of outdoor F&B advertising was greater among middle and high schools with a majority Hispanic population (compared with low)	Within half a mile radius of 34 middle and 13 high schools in central Texas, USA. (5653 F&B ads)	Cross-sectional, observational, content analysis	Researchers noted descriptive information and took photos of every F&B ad. Ads were coded as freestanding or establishment and themes such as price, deals/value meals. Demographic information was used to allocate schools as ‘Hispanic majority’ or ‘non-Hispanic’	Freestanding (signs on gas pumps, sidewalks, A-frames, banners, billboards) and establishment ads (any signage directly placed on windows/walls of establishments) promoting foods or beverages.	*PROGRESS*: Schools with 60% or more Hispanic students were exposed to significantly more establishment ads (B = 59.7, SE = 23.04, *p* = .02), and total ads (B = 94.9, SE = 39.49, *p* = .02), but not significantly more freestanding ads (*p* = .07) compared to youth attending schools with less than 60% Hispanic students. Mean ads around schools: > 60% Hispanic students, M = 172.8, < 60% Hispanic, M = 77.8. *Power:* Schools with majority Hispanic students had significantly more price promotions (B = 17.9, SE = 8.93, *p* = .05)
Isgor et al. (2016) [56]	To examine the prevalence of outdoor F&B ads on the exterior and property of retail food outlets in relation to community demographic and socioeconomic characteristics in a nationwide sample of communities in the U.S.	Outside 1684 supermarket/grocery stores and 6337 limited service stores in a sample of 469 communities in the US.	Cross-sectional, observational, content-analysis	Researchers observed the chosen stores through counting exterior ads. Ads with F&B price promotions, fresh fruit and vegetables and regular soda were tallied.	Ads on store building’s exterior and/or store properties. A minimum of 8.5″ by 11″.	Exterior F&B ads were present at 58.6% of supermarkets and 73% of limited service stores. Fruit and veg ads were more frequently displayed by supermarkets than limited service stores (26.2% vs 2.7%). Regular soda ads more frequent in limited service stores (41.3% vs. 15.8%) than supermarkets. *Power:* Price promotion ads were more frequent in limited service stores (54.3% vs. 39.9%). *Power & PROGRESS:* Price promotion ads more frequent at supermarkets in non-Hispanic black communities (58.7%) than non-Hispanic white (39.6%). This was no longer significant after controlling for household income. Supermarkets in low-income communities were 92% more likely to have price promotions. *PROGRESS:* F&B ads and soda ads were significantly more prevalent at supermarkets in majority non-Hispanic Black (75.9, 30.5%) and majority Hispanic (69.3, 24.4%) communities compared to majority non-Hispanic white (57.1, 14.3%). This was no longer significant after controlling for household income. F&B ad prevalence was higher in low-income communities. Supermarkets located in low-income communities had 1.7 times higher odds of displaying any F&B ads (95% CI = 1.11–2.61), and were 2.14 times more likely to have regular soda ads (95% CI = 1.32–3.47). Limited service stores – more fruit and veg ads in majority Hispanic (4.1%) communities compared to majority non-Hispanic white communities (2.4%). More soda ads in non-Hispanic black (48.1%) than non-Hispanic white (42.2%). Being a limited service store in a low-income community was associated with 47% lower odds of displaying fruit and vegetable ads (95% CI = 0.3–0.92).
Nelson et al. (2020) [76]	To identify and describe the food promotion environment to gauge the extent to which consumers may be exposed to low-nutrient F&B messages *in retail and restaurant contexts (Study 1)* and out-of-home advertising (Study 2).	132 F&B ads in high-traffic locations in Kingston, Jamaica	Cross-sectional, observational, content analysis	Researchers captured all “publicly viewable advertising” on all surfaces in specified locations. F&B ads coded by food category (everyday, select carefully and occasionally), type of ad, branding, localisation of messages, spokespersons, display of food.	On-premises signage, billboards, bus shelters, transit	Approximately 26% of ads (127 out of 484) were for F&B. 66.6% of all F&B were “less healthy” or occasional (without alcohol, calculated from available data), 14.5% were healthy. 34.8% of food ads (including alcohol) were for fast food franchises, 12.9% were soft drink brands. “Almost 30%” of food ads featured a soda. *Power:* Emotional appeals (happiness, fun or excitedness) were used in 50% of soft drink ads. Value appeals (12.1%) and taste appeals (12.1%) were used, “often” by fast food restaurants. “Very few” everyday products used emotional, value, or taste appeals. *PROGRESS:* “More than half” of fast food ads and 82.4% of soft drink ads were US brands. 21% of ads using a local approach (Jamaican/Caribbean words, focus on history or culture) were classed as everyday, whereas 15.67% of ads using a global approach (not tailored to local market) were classed as “everyday”. *Note: additional food data was not extracted due to being combined with alcoholic beverages*
Ohri-Vachaspati et al. (2015) [57]	To examine the extent of child-directed marketing (CDM) inside and on the exterior of fast food restaurants.	6716 fast food restaurants across 46 states, USA	Cross-sectional, observational, content analysis	Data collectors drove the streets of catchment areas to observe sampled venues and discover any venues not already identified. CDM inside and on exterior of restaurants was recorded. Community data obtained from American community survey. Both chains and non-chains were observed.	Child directed marketing - ads with cartoon characters, ads with movie, TV or sports figures, ads of kids’ meal toys, exterior play area	*Power/Demographic*: 16.8% of restaurants had any exterior CDM. 5.2% used exterior ads with cartoon characters, 2.7% used exterior ads with sports figures or TV/movie stars, 5% used exterior ads for kids’ meal toys. *PROGRESS:* Being located in middle income (compared to high) and black communities were “marginally significantly” associated with increased odds of any CDM. Rural neighbourhoods had 40% greater odds of CDM than urban.
Sousa et al. (2020) [77]	To assess the scale of billboard advertising of food and drinks in Maputo	149 billboards on streets in Maputo, Mozambique (including 500 m buffers around schools)	Cross-sectional, observational, content analysis	All billboards were photographed and location recorded using GPS. Content analysis documented language used, foods were classed as core or non-core, companies classified as national/multinational and ads classed as children-targeted or general. Main emphasis also reported (e.g. pleasure, taste...) Any non-core ads near to schools (500 m) was recorded.	All permanent billboards with outdoor static advertising	21.5% (*n* = 32) of billboards advertised F&B. Of those, 78.1% were non-core. Frequently advertised foods were soft drinks (25%, *n* = 8) and chocolate or chocolate cookies (21.9%, *n* = 7). 6 billboards advertised non-core foods from local companies. Proximity to schools was frequent (median of 4 schools close to a billboard for non-core foods). Median walking distance from schools was 240 m. *Power:* Non-core food products emphasised pleasure, taste and satisfaction related to consumption (65.6%). *PROGRESS:* Most ads (71.9%) used Portuguese *Demographic:* Non-core foods targeted the general population (not just children) (90.6%), the 3 (2%, calculated from available data) billboards targeting children advertised non-core products rich in sugar, and fat.
Vandevijvere et al. (2018) [78]	To analyse, for the first time, the extent and nature of food marketing around a large sample of New Zealand schools and formulate implications for policy.	30,494 F&B ads within 500 m buffers around a convenience sample of 950 schools across New Zealand. (14,310 ads in subsample)	Cross-sectional, observational, content analysis	All streets within buffer zones were surveyed and food ads were gathered. For each school, the number of food ads and junk food ads was recorded. A sample of schools (*n* = 535) underwent a more detailed assessment where pictures were taken and analysed. Foods were coded as junk food/non-junk food, permitted/non-permitted, and everyday/sometimes/occasional. Presence of promotional characters and premium offers within ads were also recorded.	Billboards, posters, free standing signs, neon signs, stickers, electronic boards, banners, bus shelter signs and signs on outdoor furniture, bridge/awning signs and painted buildings. Signage (for store identification) was excluded unless also acting as promotional material.	79.6% of schools had food marketing nearby. 78.3% had junk food marketing. The median number of junk food ads was 9 for all schools and 11 for urban schools. For the subsample, 9.4 ads per km (11 per km urban; median) were not permitted to be marketed to children. Maximum number of ads not permitted was 468.5 per km^2^. 55.2% of ads were for junk food, 65% not permitted, 61% classed as occasional. Most frequent categories included SSBs (20.4%) fast food such as pizzas and burgers (19.2%), sweet bread, biscuits, cakes, muffins, pies and pastries (8.8%), ice cream and desserts (8.5%) and diet soft drinks (4.3%). 5.3% were for brands/companies rather than specific products. *Power:* 28.1% of ads included a premium offer, 3.5% used promotional characters. *PROGRESS:* Median proportion of junk food ads was significantly higher (*p* < 0.001) around schools with the highest compared to the lowest number of socio-economically deprived children. Median number of junk food ads was not significantly different by level of deprivation.
Velazquez et al. (2019) [79]	To describe the prevalence and characteristics of F&B ads surrounding public elementary and secondary schools in a large Canadian city.	800 m buffer areas around 25 schools in Vancouver, Canada (653 ads)	Cross-sectional, observational, content analysis	Surveyors visited each major commercial street within buffers, documenting locations and photographing all food stores and food related promotions. Ads were coded according to main purpose, food category, marketing techniques and whether materials were professionally made. SES assessed by Vancouver Area Neighbourhood Deprivation Index.	Posters or other physical materials, images related to food to relay information or increase awareness and store signage	84% of schools (21 out of 25) had at least one F&B ad. 40% had fewer than 10, 20% had 50+. 76.6% of ads were located on food outlets. 5.7% of items were for “sell most” products, 24% “sell sometimes” and 45.6% “do not sell”. Most frequent products were mixed entrees such as pizzas and burgers (20.7%), “other” beverages including soft drinks (19.4%) and milk and alternatives (19%). 44 ads featured fruit or vegetables, almost half of which (*n* = 21) were classed as “sell sometimes” or “do not sell” (due to containing added sugar). *Power:* Cartoons/celebrity characters were present in 2.8% of food ads and premium offers present in 9.8%. 74.3% of ads included branding associated with a provincially or nationally recognisable company. *PROGRESS:* Most deprived neighbourhoods had more branded ads (56.2% vs 41.9%) and food pictures (30.1% vs 22.2%). The 8 school neighbourhoods with highest deprivation had a higher proportion of “do not sell” products (49.7%) compared to least deprived (38.9%). Areas of high deprivation also had a higher proportion of “sell most” ads (7.9% vs 3.6%). Least deprived schools had a greater proportion of “sell sometimes” ads (23.4% vs 20.3%), and brand ads (rather than specific products (34.1% vs 22.1%)). Results do not support a socioeconomic gradient (after adjusting for school type/store makeup), however there were significantly more “sell most” ads around schools in the most deprived compared to least deprived areas (*p* < 0.05).

Abbreviations: *F&B* Food and beverage, *Ads* Advertisements, *SSBs* Sugar-sweetened beverages*, WHO* World Health Organization, *SES* socioeconomic status, *CDM* Child-directed marketing

**Table 3:** Studies measuring exposure to outdoor food marketing

Author(s), year of publication	Study objectives	Study setting	Study Design/ participants	Methods	Advertising mediums included	Main results relating to outdoor advertising
Adams et al. (2010) [80]	To explore differences in the prevalence of outdoor food advertising, and the type and nutritional content of advertised foods, according to an area-based marker of socio-economic position (SEP)	Bus routes and areas around shops in Newcastle upon Tyne city centre, UK. (1371 ads)	Cross-sectional, observational, content analysis	All outdoor ads within city boundaries were identified, photographed, their size estimated (m^2^) and location determined using a GPS device. Ads were classified as food or non-food and food ads grouped into six categories based on the ‘eatwell plate’ plus an additional category: other foods. Nutritional information was obtained and classified as HFSS or not (using UKNPM). Data was compared using the English Index of Multiple Deprivation.	All outdoor advertisements for any product	15% (*n* = 211) of ads were for food (20% of ad space). “Advertised foods tended to be high in fat and low in protein, carbohydrate and fibre”. 33.9% of food ad space was devoted to HFSS products. 14.7% of food ads (*n* = 31) were for the ‘KFC Buffalo Toasted Twister’. All food ads featured specific products rather than brands generally. *PROGRESS*: The proportion of ads for food was significantly smaller in the most (13%) vs. the least (18.4%) affluent tertile (*p* < 0.05) however foods advertised in the most affluent tertile were the poorest choice for health, whilst foods in the middle tertile were the best choice for health. Total food advertising space was larger in the least affluent tertile, but proportion of advertising space for food was highest in the middle tertile(24.3%). HFSS advertising space was significantly higher in the middle than in the least affluent tertile (*p* < 0.001). Little evidence that nutritional content or types of food showed consistent socio-economic trends across tertiles.
Adjoian et al. (2019) [58]	To determine if outdoor advertising density for non-alcoholic drinks, food, tobacco products, and alcohol, is associated with neighbourhood poverty or other Census-level characteristics in New York City (NYC)	Random sample of retail-dense streets in New York City (1106 sampled street segments, 953 eligible) (16,305 ads for consumable products including alcohol and tobacco)	Cross-sectional, observational, content analysis	Street segments were stratified by poverty level (determined by census tract data) and borough. Photographs were taken of street-level ads for consumable products and coded for location type and content. For each ad, coders noted the presence of (1) non-alcoholic beverages, including sugary drinks, low-calorie drinks, water or seltzer, unsweetened coffee, other drinks and unknown drinks; (2) food, including fresh produce, sweets, and other, a notation was made if ads were for fast food.	Ads located on storefronts, awnings, building walls, construction walls, newsstands, bus shelters, subway entrances, etc. were counted as well as Ads located inside an establishment but intended to be seen from the street	41.8% of consumable ads were for food and 38.6% for non-alcoholic beverages. Of all consumable products, most frequent categories were other food (29.2%), sugary drinks (27.6%), fresh produce (9.1%), sweets (8.8%) other drinks (5.3%) Food made up 54% of all product images, non-alcoholic beverages 24%. The most prevalent product images were other food (30.8%), sugary drinks (16.2%) fresh produce (13.8%), sweets (9.2%) and other drinks (2.9%). *PROGRESS*: For every 10% increase in proportion of Black residents there was a 6% increase in food images and 18% increase in non-alcoholic beverage images. Images for sugary drinks/water/seltzer and fruits and vegetables were more common in areas with a higher proportion of Black residents. Density of product images for non-alcoholic beverages and sugary drinks was higher in areas with a higher percentage of adults with less than a HS diploma. *Demographic*: Less than 1% of ads were child focused. *Note: additional food data was not extracted due to being combined with other consumable products.*
Barnes et al. (2016) [59]	1) To characterize the presence of ads for healthful and less healthful F&B 2) To describe the product placement of items for impulse buys and assess differences in marketing strategies among food stores	119 randomly selected small stores in Minneapolis, St Paul, Minnesota, US	Cross-sectional, observational, content analysis	Food retailer audits were completed on weekdays. Data collectors recorded whether images of healthful or less healthful foods were present. Healthful foods included fruit, vegetables, wholegrains. Less healthful foods defined as high calorie, low nutrient, including alcoholic beverages (all HFSS).	Images of healthful and less healthful foods on storefront doors or windows. Images defined to included brands, logos, or texts of specified food items and well-known products.	37% of stores (*n* = 44) had ads for healthful items on their exterior. 55% of food-gas marts had at least 1 healthful exterior ad, (17% for corner/small grocery stores). 11% of stores had only healthful exterior ads. *Note: additional food data was not extracted due to being combined with alcoholic beverages*
Barquera et al. (2018) [81]	To analyze the characteristics of food advertising practices around 60 elementary schools in two cities and to evaluate compliance with the Pan American Health Organization (PAHO) recommendations and the local food industry self-regulatory marketing code.	100 m buffer zones around randomly selected schools in two cities in Mexico (29 schools in Curenavaca (16 private, 13 public), 31 schools in Guadalajara (11 private, 20 public)) (278 F&B ads)	Cross-sectional, observational, content analysis	Buffer zones inspected for any commercial source of F&B advertising. Photographs taken of all food ads and ads coded by food category (soda, juices, SSBs, sweet snacks, chocolates and candies, milk and dairy products, salty/fried snacks, water, ice cream and other) and noted brand name, type of ad and use of promotions. Compliance with PAHO and PABI (industry self-regulatory code) recommendations evaluated.	Poster, banner, sticker, painting on walls, flags billboards and walls not related to stores in the buffer areas.	278 F&B ads, 69.8% of which were outside (*n* = 194), only 3 billboards were identified (excluded from analysis). *PROGRESS*: 73% of outdoor ads (*n* = 142) were identified around public schools, compared to 27% (*n* = 52) around private.
Basch et al. (2019) [60]	1) To determine the prevalence of SSB ads on LinkNYC kiosks and document the frequency containing sugar. 2) To examine whether the prevalence of SSBs varied by median income level of the census tract location.	Random selection of 100 LinkNYC kiosks (out of a possible 507) in New York city (2025 ads)	Cross-sectional, observational content analysis	Researchers stood at each kiosk for 10 minutes and coded beverage ads (type, size and flavour), zip code of each machine and corresponding median annual income for zip code using census tract data. Calories per serving of each beverage was ascertained as well as sugar content.	Digital LinkNYC kiosks - provide ability to make calls, get information and charge devices. With 55 in. screens on both sides	17.1% of ads were for non-alcoholic beverages, 59.4% (*n* = 206) of these were for SSBs including iced tea (*n* = 106) soda (*n* = 77) blended coffee drinks (*n* = 22) and flavoured water (*n* = 1). For the SSBs, the mean kilocalories and grams of sugar per serving was 149.90 and 35.04 respectively. *PROGRESS*: No significant differences observed over income quartiles. (*p* = .38)
Cassady et al. (2015) [61]	To present patterns of advertising related to the two key obesity-related behaviors, diet and physical activity, in an economically and racially diverse urban area in Northern California, and investigate whether there are disparities in the distribution of these ads by neighbourhood income and race.	Urban area of Northern California – Sacramento, USA (171 health-related outdoor ads covering 23,971ft^2^)	Cross sectional, observational, content analysis	16 zip codes were categorised as high or low income and majority ethnic group. Trained researchers used an app (droidSURVEY) to record subject, language, GPS coordinates, and a photo of each ad. Area of each ad determined. Duplicates and electronic billboards removed (multiple messages). All ads coded as unhealthy F&B or physical activity. Ads were classed as healthy if they promoted F&B encouraged by the Dietary Guidelines for Americans and unhealthy if high-calorie, low-nutrition.	Billboards, bus shelters, bus benches and posters on storefronts (electronic billboards removed)	32% of all health-related ads were F&B, (52% of total advertising area). “National brand fast food and grocery stores dominated unhealthy food advertising, partly because most of the grocery store ad areas were devoted to images of pizza and ice cream”. Ad space for beverages was equally healthy and unhealthy (5ft^2^ vs 6ft^2^ per mile) but 4x the space was devoted to unhealthy vs healthy foods (24ft^2^ vs 6ft^2^ per mile), “about half of the healthy ad space was sponsored by the government or a non-profit agency campaign”. *PROGRESS:* Low-income Latino and African American neighbourhoods had more F&B advertising (2-35x ad space) with 5x unhealthy food ad space compared to high-income white neighbourhoods and 6x that of low-income white neighbourhoods. Low-income Latino and African American neighbourhoods had 50% more ad space for unhealthy beverages. All neighbourhoods except low-income white, had more space devoted to unhealthy than healthy food ads. Unhealthy beverage ads were more dense in low-income and multiracial neighbourhoods.
Chacon et al. (2015) [82]	To describe the type of snack foods advertised to children in stores in and around public schools and assess if there was an association between child-oriented snack food advertising and proximity to schools.	Outside 55 stores around two preschools and 2 primary schools in Mixco, Guatemala (321 snack food ads)	Cross-sectional, observational, content analysis	Using Google Earth, researchers located all stores within 200 m radius of school entrances and categorised them as less than 170 m away and more than 170 m away. Researchers coded store type, number of ads inside and outside stores and those that were child-oriented.	Posters, stickers, free-standing signs, banners, painting on walls or flags.	For exterior child-oriented ads – there was a median of 1 (0–2) outside all stores, 2 (1–2) < 170 m from schools and 1 (0–2) >/170 m from school.
Dowling et al. (2020) [62]	To estimate the density of street-level sugary drink ads across the 5 boroughs of NYC and describing variation by neighbourhood.	Random sample of 953 retail-dense street segments in New York City (4356 ads)	Cross-sectional, observational, content analysis	Surveyors photographed ads along both sides of segments and coded with nutrition information to determine if beverages were “sugary drinks” (Defined as featuring pictures or logos of beverages with added caloric sweetener and > 25 cal per 8 oz. serving). Poverty determined by census tract data.	Posters, digital signs, stickers on outdoor structures such as newsstands, bus shelters and payphones.	4356 ads were identified featuring sugary drinks, with 8197 sugary drink images. On average, there were 2 images per ad. Between 2.72 and 29.91 ads for sugary drinks were observed per 1000 ft. segment. *PROGRESS:* Sugary drink ad density was 1.54 times (95% CI = 1.16, 2.04) as high for medium vs low poverty neighbourhoods and 1.66 times (95% CI = 1.26, 2.19) as great for high vs low poverty neighbourhoods. After adjusting for census tract characteristics, associations were still significant in two out of five boroughs. Associations were observed between ad density and percentage of residents with less than a High school diploma (10-unit IRR = 1.15, 95% CI = 1.04, 1.27); percentage of asian or pacific islander residents (10-unit IRR = 1.10, 95% CI = 1.01, 1.19) and the percentage of black, non-latino residents (10-unit IRR = 1.20, 95% CI = 1.14, 1.26).
Egli et al. (2019) [83]	To add to the body of knowledge on obesogenic environments for children and the utility of Google Street View in geographical health research.	800 m buffers around 19 schools in Auckland, New Zealand (2472 ads).	Cross-sectional, observational, content analysis	Two independent research assistants ‘travelled’ around streets in the 800 m buffer areas on Google Street View between March and July 2017 and took screenshots of outdoor ads. Distance from school to ads was measured using Google Maps. Foods reflecting dietary patterns associated with increased risk of obesity and dental caries in childhood, processed, energy-dense, nutrient-poor were classed as “unhealthy”.	billboards, signs, flags, banners, balloons, neon signs, stickers, and bus shelter ads that were large enough to be seen on a 15 in. computer screen; with an identifiable logo or text; and located completely or partially on public land	2474 ads identified, 29.4% could not be identified (blurry), of identified ads, 15.5% were for F&B (calculated from available data). Of total ads, the most frequent food categories were “food, other” (5.6%), “food, unhealthy” (5.4%), beverage other (2.1%), F&B other (0.9%), and SSBs (0.9%). *Demographic:* 46.6% of F&B ads were marketed to children and adults, none to children only. Of ads marketed to both adults and children, there were significantly more unhealthy F&B than other F&B (*P* = 0.001). *PROGRESS*: No significant difference in F&B advertising marketed to children by neighbourhood deprivation *Note: additional food data was not extracted due to being combined with alcoholic beverages*
Fagerberg et al. (2019) [84]	To explore the proportion of ads directly related to ultra-processed foods (including sugary drinks and fast foods) in two areas of Stockholm, Sweden with low vs. high SES	Two districts of Stockholm, Sweden. Around/in subway stations and shopping malls (Östermalm (high SES) and Skärholmen (low SES)) –4092 ads	Cross-sectional, observational, content analysis	All ads were recorded by two researchers using smartphones. Pictures were taken, and annotated by a dietician according to difficulty to annotate, location, food or not, sugary drink or not, including food promotion or not. Following categorisation, another category was created (ultra-processed food) by adding the sum of “sugary drink” and “fast food” categories.	All ads in the subway station and 50 m to the left and right on the streets outside entrances to subway. All ads in shopping mall and on streets surrounding shopping mall. Excluded - flyers/brochures, ads inside/outside moving vehicles, inside stores. Subway escalator ads excluded as they change weekly.	32.8% of ads (1341 pictures) were for food products. 65.4% of food ads promoted ultra-processed products (877 pictures). *PROGRESS:* No significant difference in proportion of food ads out of total ads by SES, however there was a significantly higher proportion of ultra-processed food ads in Skarholmen (low SES) compared to Ostermalm (high SES) (73% vs 59%, *p* < 0.001/*p* = 0.001). The higher proportion of ultra-processed ads in Skärholmen can be explained by a higher proportion of fast food ads; 65.4% vs 48.8% (*p* = 0.000; researcher 1) and 61.1% vs 36.4% (*p* < 0.001; researchers 2 + 3). There was no significant difference in the proportion of sugary drink ads: 30.7% vs 28.1% (*p* = 0.442; researcher 1) and 29.1% vs 30.6% (*p* = 0.700; researchers 2 + 3).
Feeley et al. (2016) [85]	To assess consumption of commercially produced foods including breast milk substitutes, commercially produced complementary foods and various snack food products consumed by children less than 2 years of age as reported by their mothers, and maternal recall of promotions of these products.	Outside sampled health facilities in Dakar, Senegal.	Cross-sectional survey 293 mothers of children utilizing public/faithbased/non-governmental health facilities	Mothers were interviewed about F&B consumed on the day/night prior to the interview. Data also gathered on weekly consumption, reasons for feeding, expenditure on snacks and promotional practices inside and outside health facilities for breast milk substitutes and commercially produced complementary foods and commercially produced snack foods.	Billboards	4.4% of participants reported seeing a billboard promoting breast milk substitute. 4.4% of participants reported seeing promotion of commercially produced complementary foods.
Huang et al. (2020) [86]	To determine whether there are associations between school decile, distance from school, walk and transit scores, and the prevalence of non-core F&B promotion at bus stops within walking distance from all Auckland schools.	500 m around 190 schools in Auckland, New Zealand (842 ads)	Cross-sectional, observational, Content analysis	Google Street View was used to screenshot images of bus shelter ads which were saved by school type. The shortest walking distance was calculated using the map tool in GSV. F&B were coded according to WHO Euro NPM, making them core or non-core.	Ads on each side of bus shelters large enough to be seen on a 15-in. computer screen and with an identifiable logo or text.	842 ads were identified, 25.5% promoted F&B. 50.2%) were non-core. The food categories promoted were core foods (9.7% of all ads), non-core foods (8.1%), non-core beverage (3.6%), and core beverages (3%). All ads promoting non-core F&B together were fast food, none promoted core F&B together. The number of F&B ads per 100 m increased as the distance from schools increased. *PROGRESS:* There were 702 ads around state or state integrated schools, 140 around private. The greatest proportion of ads for non-core food (33.8%), core food (32.9%), and non-core F&B (50%) were found in low deciles, followed by high deciles then medium. High decile schools had the greatest proportion of core beverage ads (40%). When high-decile areas were combined with areas around private schools, the greatest number of all F&B ads (42.3%) and non-core ads (41.7%) were found in high SES areas.
Kelly et al. (2008) [87]	To quantify the volume of and factors associated with food ads in an environment where young children are commonly found (area surrounding primary schools). We also aimed to identify the specific food products that are advertised around primary schools.	20 Local government areas in Sydney and Wollongong, Australia with high/low population density and high/low SES. 250 and 500 m radius around 40 primary schools (9151 ads, 2286 for food)	Cross-sectional, Observational, content analysis	Two primary schools randomly selected from each government area. A coder navigated the 250 m and 500 m radius around schools to identify all ads. Ad characteristics captured: type (food/non-food), description of product, distance to school, size, location and foods further into core and non-core.	Standard commercial ads (billboards, posters) temporary advertising of events, outdoor furniture, signs on buildings (name and branded product information) Signage was excluded from analysis = all signs unaccompanied by branded product information (identification of sites etc.)	19.5% of ads were for F&B, 58% of which were non-core (without alcohol, calculated from available data), 5% of F&B were core, 15% tea or coffee. Most frequently advertised foods were soft drinks (24%), coffee (15%), and ice cream and confection (14%). < 250 m from schools 69.7 out of 114.9 food ads per km^2^ (60.7%) were non-core foods (not including alcohol, calculated from data) compared to 33.5 out of 58.8 food ads per km^2^ (56.8%) 250-500 m away from schools. *Note: additional food data was not extracted due to being combined with alcoholic beverages*
Kelly et al. (2015) [88]	To identify, describe and quantify the volume of F&B ads around schools in two demographically and culturally disparate cities in Asia.	500 m around 30 primary schools in Ulaanbaatar (Mongolia; 1459 ads and Manila, The Philippines; 9687 ads).	Cross-sectional, observational, content analysis	Primary schools were randomly selected in each city. Researchers surveyed all streets within 250 and 500 m from schools. Noted distance from school, size of ad, setting, type and position, whether single or multiple foods, brand name and type. Foods were classed as core/healthy, non-core/unhealthy and miscellaneous.	billboards, posters, free standing signs, neon signs, stickers, electronic boards, banners, bus shelter signs and signs on outdoor furniture, bridge/awning signs- and painted buildings. Store signage with a product logo in addition to identification was included.	Mongolia – 1459 food ads were identified, 88% were non-core (not including alcohol, calculated from available data) 52% of non-core ads were SSBs. Coca-cola (35%) and Pepsi (10%) were the most frequent food/drink brands advertised. Philippines - 9687 food ads were identified, 78% were non-core (not including alcohol, calculated from available data). 56% of non-core ads were for SSBs. The most frequently promoted brand was Coca-Cola (32%) followed by a local soft drink manufacturer (RC Cola – 8%) *Note: additional food data was not extracted due to being combined with alcoholic beverages*
Liu et al. (2020) [89]	1) Measure and visualize children’s space and time exposures to unhealthy food advertising in public, outdoor spaces, using GPS and wearable camera technology; and 2) Test potential reduction of exposure by proposing banning unhealthy food advertising near schools, playgrounds, or in residential areas.	Wellington City, New Zealand (59,150 ads)	Cross-sectional, observational, content analysis 138 students from 16 schools aged 11–14	Participants wore cameras and GPS devices for four consecutive days. Cameras captured a 136^o^ image every 7 seconds. All images coded for food advertising exposure through content analysis. Foods classified as core or non-core based on WHO NPM. GPS data (recorded every 5 seconds) was linked to image codes	Ads in outdoor public areas	Children were exposed to a mean of 8.3 food ads every hour they spent in outdoor public areas (95% CI 7.9–8.7). 89.2% of these were unhealthy, 9.6% were healthy. Shop fronts and main streets were the “most common settings” for unhealthy food products. “Children were exposed most commonly to advertisements for fast food, sugary drinks, ice cream, cookies and confectionary.” Weekday exposures “frequently occurred during times of travel to and from school”. Public transport facilities (11 children), fresh food markets (6 children), and service station forecourts (n/a) were the least frequent exposure settings.
Lo et al. (2020) [90]	To examine the extent and characteristics of F&B promotion in Hong Kong mass transit railway (MTR) stations in districts with different SES and school density and to assess if children or lower SES audiences were specifically targeted by advertising.	8 stations of the Mass transit railways in Hong Kong (8064 ads)	Repeated cross-sectional, observational, content analysis	8 stations selected and classified by SES and school/non-school zone. Stations were visited on three occasions, photos and videos of all ads were taken - for digital promotion, an average of 3-minute videos were taken. F&Bs were categorised into three groups (core and healthy, non-core and unhealthy and seasonal and special) The number of F&B in each ad was documented and healthiness of each product reported.	All ads located in the MTR station area (escalator, platform, trackside, shops, paid and unpaid area and outside of stations)	10.6% of ads were food-related (calculated from available data). This was higher in seasonal periods (15.3 and 13.1% vs 7.3%). 53.5% of food ads were for non-core foods (excluding alcohol, calculated from available data), 43% were core and healthy, the remaining were seasonal and special (2.5%). Most frequently advertised food products were: sweet breads, cakes and pastries (18.3%), meat and meat alternatives (17.3%), grains and bread (9%), SSBs (8.8%) and processed meat/alternatives (6.8%). Stations in school zones had a significantly higher proportion of ads for processed grains (2.7% vs 7%), processed meat and meat alternatives (9.1% vs 4.9%), high-sugar snacks (1.5% vs 0.1%), sweet breads (23.2% vs 14.3%) and seasonal food/items for special purposes (1.7% vs 0.7%) and fewer ads for fruit and fruit products (1.5% vs 3.2%) and Chinese dried seafood and tonic (0% vs 1.9%). *PROGRESS:* In high SES areas, 41.7% of food ads were core and 54.9 non-core (calculated without alcohol) compared to 47.1% core and 49.5% non-core (calculated without alcohol) in low SES. High SES stations had a higher proportion of ads for processed meat/meat alternatives (7.9% vs 4%) and Chinese dried seafood and tonics (1.5% vs 0%), and fewer ads for fruit/fruit products (1.6% vs 4.6%), milk/dairy (0.5% vs 1.9%) and processed grains (0.9% vs 3.2%). *Note: additional food data was not extracted due to being combined with alcoholic beverages*
Lowery & Sloane (2014) [63]	To examine associations between the content of outdoor advertising and neighbourhood ethnic/racial and socioeconomic composition to see whether particular communities disproportionately host harm.	Sign districts in Los Angeles (legislation limits outdoor ads to 21 commercially zoned sign districts)	Cross-sectional, observational, content analysis	7/21 sign districts were selected using census tracts within 500 ft. of each regional center. Data on the area of each sign district, street length and number of intersections obtained from City of Los Angeles Department of City Planning. Photographs of ads were taken monthly for 7 months. Harmful content coded into 5 categories (encouraging addictive behaviours (alcohol/tobacco), violence, unhealthy eating, unsafe environments for women and content inappropriate for children)	Billboards (digital and conventional)	Most frequent unhealthy food ads were for “fast food options (hamburgers, fried foods) and advertisements for soft drinks, flavoured beverages and candy”. *PROGRESS:* Ads featuring unhealthy food options were most prevalent in the African American community in Baldwin Hills (18.6%) and the Latino community more densely populated with young people (12.7%)
Lucan et al. (2017) [64]	To assess all print advertising in all stations of a subway system for large urban county. The goal was to determine how placement of ads for foods and beverages related to subway ridership and to the demographics, dietary intake, and prevalence of diet-related conditions in the residential areas surrounding stations.	68 Subway stations in the Bronx, New York, USA (1586 ads, 284 distinct)	Cross sectional, observational, content analysis	Researchers rode all subway lines, assessed all print ads and decided whether they were targeting certain groups (minorities or youth). F&B ads were recorded as “more healthful” or “less healthful”. Census data was gathered, and ridership data was collected from the Metropolitan transport authority. Self-reported fruit and vegetable consumption and presence of diet-related conditions was provided by the city health department	Wall posters, free-standing billboards, or other signage (on trash cans, receptacles, turnstiles, station clocks or benches)	39.7% (27/68) of stations had food ads. No ads promoted “more-healthful” foods or beverages, however 12 distinct ads (27.9%) pictured items classed as “more-healthful”. Distinct ads: 10.9% (*n* = 31) were for F&B (calculated without alcohol), **39.3%** were less healthful (calculated). There were 5 distinct ads for non-alcoholic beverages and 12 for food. Including duplicates: 7.8% (*n* = 124) of ads were for F&B (calculated from available data), 39.3% were less healthful. There were 24 ads for non-alcoholic beverages and 40 for food. *Demographic:* 26.1% of stations had less healthful food ads directed at youth. *Note: additional food data was not extracted due to being combined with alcoholic beverages*
Maher et al. (2005) [91]	To examine the extent and content of outdoor food ads and food availability from outlets in the vicinity of secondary schools.	1 km radius around a convenience sample of 10 rural and urban high schools in New Zealand (1408 ads)	Cross-sectional, observational, content analysis	School neighbourhood SES obtained through Ministry of Education classification system. Researchers walked around the area, recorded ad info and took a photograph of all F&B ads. Food ads classified according to food type, products with desirable and undesirable characteristics labelled as healthy for a conservative approach	Billboards, neon signs, posters, stickers, free-standing signs, banners, painted buildings, bus shelter ads, flax and images in shop windows designed for viewing outside. Ads on buses/delivery vehicles excluded.	Approximately 57% of ads surrounding schools were for food products (excluding alcohol, calculated from available data). The most frequent food categories were soft drinks (21.6%), healthy foods (e.g. bread, milk; 18.8%), frozen confectionary (16.2%) and savoury snacks (11.4%). The most frequent food brands advertised were Coca-Cola (17.6%), Tip Top ice cream (10.4%) and Meadow Fresh dairy foods (3.9%). *PROGRESS:* There was a significantly greater proportion of ads for takeaway outlets (RR 1.54, *p* < .001) and fast food outlets (RR 1.67, *p* < .001) in higher SES areas. Staple food ads were more frequent in low SES areas (RR 2.04, 95%CI 1.54 to 2.69, *p* < .00001). The proportion of frozen confectionary foods ads was higher in rural than urban neighbourhoods (*p* = 0.048) *Note: additional food data was not extracted due to being combined with alcoholic beverages*
Moodley et al. (2015) [92]	To investigate the density of outdoor SSB advertising and the number of formal and informal vendors selling SSBs in a transforming, historically disadvantaged urban setting of South Africa.	5 areas of Soweto, Johannesburg (145 ads)	Cross-sectional, observational, spatial analysis	Researchers walked or drove through each street in the study area. Data was collected on location (GPS) and type of ads and food vendors. Photos were taken of all ads. Size of ads estimated (S/M/L). GPS used to create spatial point patterns and assess the association of points to the nearest school.	Billboards, bus stop ads, signs placed along the sidewalk, urban art on streets or buildings, large posters and signage for restaurants or food vendors (taxis and buses excluded)	145 SSB ads were identified. Ads were frequently located outside houses (53%), at small shopping centres (15.9%), on the street (13.8%), at schools (9%) or other buildings (4.8%). Frequent formats were shop signs (54.5%), posters (16.6%), painted ads (10.3%), school signs (8.3%) and bus stops (4.1%). 62% of branded SSB ads were part of a display sign for a shop. 13/28 primary and secondary schools displayed SSBs on branded school signs. There was an increase in SSB ads with decreasing proximity to nearest school (RI −2.7, 95%CI = 2.48–1.82).
Olsen et al. (2021) [93]	(1) Categorise the content of ads at bus stops across a large and varied geographical area. (2) Explore associations between the socio-spatial distribution of bus stop ads (3) Test for associations between specific categories of unhealthy commodity ads in the local area surrounding schools (4) Calculate children’s ‘real’ exposure to bus stop ads using individual mobility data of Scottish children.	1845 bus shelters in the central belt of Scotland, UK (including the two most populated cities - Glasgow and Edinburgh) (3123 ads)	Cross-sectional, observational, content analysis 229 children (aged 10–11) who took part in the SPACES study in the central belt of Scotland	CANVAS Google Street View software was used to identify bus stops and the most recent image captured of bus shelters was audited. Bus stops were assigned deprivation ranks using the Scottish Index of Multiple Deprivation. School locations were plotted on a map and 100 m, 200 m and 800 m road and path buffers were created around each school. Children were asked to wear accelerometer and GPS to measure “real exposure” over 8 consecutive days. Bus shelter ads were categorised as unhealthy food and/or drink, SSBs, alcohol, e-cigarette product, gambling	Bus shelters	Unhealthy commodity ads were frequently for fast food (15.3%), confectionary (6.8%), SSBs (3.8%), water (3.3%) and ice cream/frozen desserts (2.3%). It was unlikely that unhealthy products were advertised within the school environment (100 m–800 m distance). Children living in urban areas had greater exposure to unhealthy food (coef: 1.29, 95%CI 1.04–1.31) and unhealthy food and drink (coef: 1.29, 95%CI 1.04–1.31) than those in rural areas. *PROGRESS:* There did not appear to be a social relationship between deprivation and ad location or type. Measures of real exposure found that children residing in more deprived areas had more contact with the transport network and so were significantly more exposed to unhealthy foods (coef: 1.18, 95%CI 1.06–1.31) and unhealthy food and drink (coef: 1.18, 95%CI 1.0–1.31).
Palmer et al. (2021) [94]	To develop a deep learning workflow to automatically extract and classify unhealthy ads from street view images.	Three areas of Liverpool, UK of differing levels of deprivation - City Centre, North Liverpool and South Liverpool (10,106 ads)	Cross-sectional, observational, content analysis	One researcher cycled on major roads in areas of interest with a camera attached recording images at 0.5 second intervals. Categorised areas (e.g. billboards) were extracted through seamless scene segmentation.	Billboards, company logos and store front signs.	10,106 ads identified in total, 13.2% (*n* = 1335) were for food. Following duplicate removal, 873 food ads remained. *PROGRESS:* “Larger proportions” of food ads were found in deciles 1–6 (compared with 7–10). “Largest proportions” of food ads can be found within cosmopolitan groups, multicultural metropolitans and hard-pressed living groups.
Parnell et al. (2018) [95]	To assess the volume and type of unhealthy bus shelter ads near kindergartens, primary and secondary schools in five local government areas in Perth, Western Australia, and assess changes with seasonality.	37 Bus shelters 500 m around schools in 5 local governments (all high SES) in Perth, Western Australia – (293 ads over four audits)	Repeated cross-sectional, observational	Researchers visited each shelter, recording the company, product and location. Each shelter ad was photographed. This was completed four times over the course of a year. Ads were coded as healthy, moderate or unhealthy by a nutritionist using guidelines from the Australian government.	Bus shelters	Across the four audits, 21% of bus shelter ads were for unhealthy food/non-alcoholic beverage (62/293). This was 67.4% of all unhealthy ads (including alcohol and gambling). Less than 1% of ads promoted a healthy product. There was no significant variation in the volume of unhealthy advertising by season. Most frequent unhealthy food products were fast food meal deal (14.5% of unhealthy F&B ads) fast food burger (12.9%), chocolate (11.3%), ice cream (9.7%), fast food chicken (6.5%) and fast food hash brown (6.5%).
Pasch & Poulos (2013) [65]	To document and describe F&B advertising around schools	1600 m around 4 middle schools in Austin, Texas, USA (563 ads)	Cross-sectional, observational, content analysis – pilot study	Data collectors documented the location and photographed all F&B ads (created the Outdoor Media Direct Observation Tool (DOT) on app FileMakerGo. Also documented ad type, category and description	All free standing, located on something other than a building, or billboards. Establishment advertising included ads directly attached to the building.	75.9% of F&B ads were directly attached to establishments, 24.2% free standing. Ads were most frequently located on or at convenience stores/gas stations (median of 77 ads per school). There was a median of 8.5 street side ads per school. There was a median of 22 ads per school associated with restaurants, particularly fast food (13 per school).
Pinto et al. (2007) [96]	To describe billboard advertising of F&B in Maputo, Mozambique.	9 busy downtown avenues in Maputo, Mozambique (707 billboards)	Cross-sectional, observational, content analysis	Researchers visited the chosen avenues and described billboards found. Items advertised were classified by 2 researchers as alcoholic beverages, soft drinks, fast food and non-fast food.	Billboards	10.7% (76/707) of ads advertised: soft drinks (*n* = 16), fast foods (*n* = 33) and non-fast foods (*n* = 27). Rice was the most frequent non-fast food item advertised (60%). All fast foods were products of international companies. *PROGRESS:* Soft drinks accounted for 15.3% of ads downtown and 16.1% in the suburbs. Non-fast food was more frequently advertised in the suburbs (48.4% vs 16.7%) and fast foods more frequently downtown (41.7% vs 9.7%), thought to reflect targeting of wealthier subjects - Pearson x(2) (3) = 15.2, *p* < .01). *Note: additional food data was not extracted due to being combined with alcoholic beverages*
Poulos & Pasch (2015) [66]	To provide a detailed description of the development of a tool for documenting and describing primary data collected on outdoor F&B advertising and F&B outlets. Furthermore, inter-rater reliability of the tool for documenting F&B ads and outlets will be determined.	½ mile radius around 34 middle and 13 high schools in central Texas, USA (732 ads)	Cross-sectional, observational, content analysis	Researchers created maps and driving routes covering all streets. An app was used to record ad information, photographs and GPS. Products assessed included F&B, alcohol and tobacco.	Billboards, street side sign, Directory, a-frames, banners (e.g. on entrance to shopping centre) and any other sign or establishment advertising food or beverages. Signage directly placed on windows or walls of an establishment	499 establishment ads were identified, 233 other ads. Urban schools had more ads in surrounding areas than suburban schools (229 vs 8).
Puspikawati et al. (2020) [97]	To survey areas around gathering places for children and adolescents (schools and other facilities) to identify outdoor F&B ads that may be risk factors for obesity, diabetes and CVD, in order to provide evidence for policy making.	Surabaya, the capital city of East Java (urban) and Banyuwangi district (rural), Indonesia. 0-500 m from gathering places for children and adolescents. (570 F&B ads in Banyuwangi, 960 in Surabaya)	Cross-sectional, observational, content analysis	Data collectors walked or rode a motorbike around all roads and alleys in chosen locations (0-100 m, 100-300 m, 300-500 m from gathering places) and recorded location and photographs via an app. All F&B ads were surveyed. Nutritional information of products was obtained from the packaging or internet and foods were classed as unhealthy (if containing high levels of fat, salt or sugar)	Banners, posters, billboards, video ads, outdoor ads on shops and stalls, stickers merchandise and any other items containing F&B ads	In Banyuwangi, 39.8% of F&B ads were for unhealthy foods and 1.8% healthy. SSBs were frequent (47.2%). In Surabaya, 28.2% of F&B ads were for unhealthy foods and 1.8% healthy. SSBs were frequent (46.1%). In both areas, density of unhealthy food, healthy food and healthy beverage ads increased closer to the facilities for children and adolescents (unhealthy beverages were combined with alcohol) *Note: additional food data was not extracted due to being combined with alcoholic beverages*
Richmond et al. (2020) [98]	To examine the number and type of food ads to which children are exposed when using public transport or walking to school in Sydney, Australia.	21 schools in the greater Sydney area, Australia (762 ads)	Cross-sectional, observational, content analysis	Theoretical train, bus and walking routes were planned for a one-way trip to each school using Google Maps. Researchers travelled the routes and recorded all visible ads. Advertised foods were categorised as core, discretionary or miscellaneous	Ads greater than A4 size that appeared in outdoor spaces - in train stations, and on buses, bus shelters and telephone posts (those on shop fronts and sandwich boards were not included	Approximately 30% of ads were for F&B (calculated from available data). The majority of foods were discretionary (69%, calculated without alcohol). Most frequently advertised food products were fast foods (23%), sugary drinks (17%) and snack foods (16%). *PROGRESS*: There was no significant difference in the rate of core ads across schools in high and mid/low deciles. *Note: additional food data was not extracted due to being combined with alcoholic beverages*
Robertson et al. (2017) [99]	To explore the socioeconomic patterning of food advertising at bus stops in Edinburgh, UK.	290 bus shelters in Edinburgh, UK (562 ads)	Cross-sectional, observational, content analysis	An app was used to record data on GPS coordinates, type of area, details about F&B product advertised, and price/special offers. Scottish Index of Multiple Deprivation was used to identify bus stop area level of socioeconomic deprivation.	Bus shelters	Advertised food categories include fast food outlets (39%), confectionary, coffee, food stores and fruit juice. There were no ads for fruit or vegetables (besides juices), water or low sugar beverages. PROGRESS: Across all food categories, there were no associations between increased prevalence of these ads and deprivation level. “for example, fast food outlet advertisements were no more likely to be present in lower versus higher socioeconomic areas (B = 0.248, 95% CI: −0.082, 0.578, *p* = 0.140). *Note: additional food data was not extracted due to being combined with alcoholic beverages*
Sainsbury et al. (2017) [100]	To examine the extent of F&B advertising on the Sydney metropolitan train network, and to assess the nutritional quality of advertised products	178 train stations on the Sydney metropolitan train network, Australia – (6931 ads)	Repeated cross-sectional, observational, content analysis	All stations on the Sydney metropolitan network were surveyed for ads and grouped by SES according to postal area. Data was collected over one week in summer and one week in winter. All ads were recorded and photographs taken, information collected included on product name and description, brand/company, location and format. All were classed as food or non-food and food ads classed as core, discretionary (not for daily consumption) or miscellaneous (e.g. tea or brand only). These groups were further divided into 32 product subcategories. Ads were also coded into size categories.	Any commercial billboard, poster, flyers, branded furniture, vending machine and experiential displays promoting a product, service or brand - specifically the train station concourse, station platform, cross-track billboards or any external ads designed to be seen by commuters standing on the platform or entering/exiting the station. For rotating billboards, all ads shown within one complete rotation were recorded	Approximately 25.9% of ads (1795/6931) promoted food and non-alcoholic beverages. There was no significant difference between seasons. The majority of F&B ads were for discretionary products (78.3% vs 8%, without alcohol, calculated from available data), with more discretionary foods (e.g. biscuits) in summer (*p* = 0.01) and more core foods (e.g. vegetable soup/water) in winter (*p* = 0.04). The most frequent food categories advertised were Snack foods (25%) SSBs (23%), intense sweetened beverages (18.7%), bottled water (6.8%) and high fat savoury biscuits, sweet biscuits, cakes, muffins, pastries and pies (3.8%). Just over 50% of food ads were on vending machines. Coca-Cola contributed 10.9% of F&B ads, PepsiCo 6.5%. 6.3% of F&B ads were brand only. *PROGRESS*: There was no significant difference in the number of core ads between SES groups *Note: additional food data was not extracted due to being combined with alcoholic beverages*
Settle et al. (2014) [101]	To examine the prevalence of outdoor food advertising at tram, bus and train public transit stops across the least and most socioeconomically disadvantaged areas of Melbourne, Australia	20 km radius of Deakin university, Geelong, Australia – 20 suburbs (10 from most and 10 from least disadvantaged areas (233 ads over 558 transit stops)	Cross-sectional, observational, content analysis	Auditing was undertaken by a single person, who visited each of the transit stops. Data collectors classified foods ads into 9 categories and recorded whether they were brand or food ads as well as location of ads, if any ambiguity, photos were taken and additional information recorded. Population density obtained using census data.	All train stations and bus and tram stops where a shelter was present	Ads were identified for cold beverages (including alcohol), hot beverages, snack foods, fast food, dairy and other food. There were no ads for cereal or fruit/vegetable items. *PROGRESS*: Broader product categories did not differ except for hot beverages (there were more in least-disadvantaged areas - 13% vs 2%, *p* = 0.004). 37% of ads were for convenience stores in least disadvantaged areas (compared to 15% in most disadvantaged, *p* = 0.026). 48% of ads for stores were for fast food restaurants in most-disadvantaged areas (compared to 19% in least-disadvantaged, *p* = 0.003). The proportion of ads for local restaurants was not significantly different by level of disadvantage. Ads for diet soft drink were more frequently observed in the least-disadvantaged areas compared to the most-disadvantaged areas (57% vs. 25%, *p* = 0.002). Ads for flavoured milk (8% vs 25%, *p* = 0.028) and fruit juice (2% vs 20%, *p* = 0.004) were more frequent in most disadvantaged areas. *Note: additional food data was not extracted due to being combined with alcoholic beverages*
Signal et al. (2017) [102]	To examine the frequency and nature of New Zealand children’s everyday exposure to F&B marketing across multiple media and settings. Marketing exposure was examined by SES and ethnicity.	Wellington region of New Zealand	Cross-sectional, observational, content analysis 168 year 8 children (aged 11–13)	Following four days of exposure, cameras were collected and images downloaded (images captured every 7 seconds). Children reviewed and deleted any photos they wished to before researchers viewed them. Age and BMI of children was obtained. Foods were classified as recommended or not recommended to be marketed to children (WHO NPM)	Street, shop front, shopping mall, private transport, public transport facility, onboard public transport and other retail. Images in convenience stores/supermarkets were excluded	In outdoor spaces, children were exposed to an average of 0.9 core F&B ads per day (7% of total) and 8.3 non-core F&B ads per day (30.4% of total). The most frequent advertisements were shop front (3.6 per day (core and non-core)) followed by street (2.2 per day). *Note: additional data was not extracted due to being combined with non-outdoor ads*
Timmermans et al. (2018) [103]	To explore and define socio-economic (SES) differences in urban school food environments in The Netherlands	400 m buffers around 21 secondary schools in Utrecht, the Netherlands (350 ads)	Cross-sectional, observational, content analysis	Researchers visited the buffer areas. For every ad, a picture was taken with a GPS tag. Food ads were categorised into ready to eat/vs foods that need to be prepared and then categorised into healthy/unhealthy. Neighbourhood SES was obtained from The Netherlands Institute for Social Research	Wall posters, banners, bus-stop ads, flags, free-standings signs. Stationary delivery vehicles promoting retail outlets, food brands, products or meals were included. Shop windows were excluded	40.95% of ads contained food products (143/350). There were on average 6.81 ads per school, however only 14/21 schools had ads in surrounding areas (with an average of 11.14 ads per school), 7 schools had none. 58% of ads were classed as unhealthy (13.7% healthy). The most frequently advertised food categories were other - not ready to eat (26.6%), savoury pastries (13%), SSBs (11.2%), hamburgers/kebabs (9.94%) and sweet and savoury snacks (7.5%). *PROGRESS*: SSBs (22.5% vs. 6%, *p* = 0.02), hamburgers and kebabs (22.5% vs. 7.7%, *p* = 0.04), diet soft drinks (7.5% vs 1.1%, *p* ≤ 0.05), vegetable snacks (10% vs 2.2%, *p* ≤ 0.05) and dairy with no added sugar (7.5% vs 1.1%, *p* ≤ 0.05) were more frequently advertised in lower SES school environments. No food groups were significantly more advertised in high SES school environments.
Trapp et al. (n.d.) [104]	Generate robust evidence describing, benchmarking and quantifying the volume and nature of all outdoor food advertising within a 500 m radius of Perth schools	500 m buffers around 64 schools in Perth, Australia, (5636 ads)	Cross-sectional, observational, content analysis	Buffer areas were scanned on foot for all outdoor ads. Tablets were used to enter data and photos of ads as well as geolocation to determine distance to school boundary. Ads were classed as small, medium or large and food or non-food. Food ads classed into core, non-core, miscellaneous and branding only.	Billboards, posters/banners, free-standing signs, painted walls, digital/LED billboards, Merchandising - outside food shops, non-food shops, road, building, bus shelter or train station	1993 food products were advertised across 1708 food adverts (total includes alcohol). Unhealthy foods were featured 1444 times, healthy foods 315 times and miscellaneous 234 times. Most frequently advertised foods were fast food (pictured in 14% of food ads), SSBs (13%), tea and coffee (11%), high fat/salt meals (10%) and ice cream and iced confection (7%). K-12 schools had a significantly higher proportion of healthy foods within 250 m (60%) compared to primary (33%) and secondary (30%). *PROGRESS:* Schools located in low SES areas had a significantly higher proportion of unhealthy food (excluding alcohol) ads within 250 m (40%) compared to high SES areas (30%). *Note: additional food data was not extracted due to being combined with alcoholic beverages*
Walton et al. (2009) [105]	To document the community food environment surrounding case study primary schools, and second, to consider whether aspects of the community food environment impact on the food environment within schools.	2 km buffers around 4 schools in the Wellington region of New Zealand (79 ads)	Cross sectional, case comparison, content analysis	Data collected by driving and walking the streets buffer areas. Locations of ads were captured and a photo taken at each location. Advertised products categorised as “everyday”, “sometimes” and “occasional”. Deprivation level defined by School’s decile rating (from Ministry of Education).	Attached to food outlets	63.3% (50/79) of ads were for “occasional” products, 22.8% (18) “sometimes” and 13.9% (11) “everyday”. *PROGRESS:* School 4 (mid-low deprivation) had no ads. School 1 (highest deprivation) had 28 total ads, 20 were “occasional”, 2 “everyday”. School 2 had 16 ads, 8 “occasional”, 1 “everyday”. School 3 (mid-high deprivation) had 35 ads, 22 “occasional” and 8 “everyday”, 5 “sometimes”
Watson et al. (2021) [106]	To compare six nutrient profiling models for suitability in food marketing to children regulation	Public transport property in Sydney, Australia (946 ads)	Cross-sectional survey	Researchers visited six transport hubs, photographed and recorded F&B advertising. Ads were classified into identifiable F&B, master brand, generic F&B, incidental F&B (non-food ad). Foods were classified as eligible or ineligible to be advertised	Advertising on buses and at train stations - on platforms, concourses and around entry gates.	964 ads were photographed, 176 unique. 150 F&B ads with 220 unique products were analysed. Of identified products (*n* = 210), 175 (83%) were ineligible to be advertised 35 (17%) were eligible (by COAG - Council of Australian Government Health Council guide). The most frequent unhealthy food categories were unhealthy meals (38.6%), SSBs (19%), dessert/ice cream (8.6%), savoury snacks (6.2%) and confectionary (3.8%). The proportion of foods eligible to be advertised ranged from 10 to 28% depending on model: Health star rating, WHO western pacific, WHO Europe and NOVA food classification system.
Yancey et al. (2009) [67]	To examine whether African Americans, Latinos, and people living in low-income neighbourhoods are disproportionately exposed to ads for high-calorie, low nutrient–dense F&Bs and for sedentary entertainment and transportation and are relatively underexposed to advertising for nutritious F&Bs and goods and services promoting physical activities.	Selected zip codes in Los Angeles, Austin, New York City and Philadelphia. (2233 ads)	Cross-sectional prevalence study	Researchers used GPS and digital cameras to create a record of outdoor ads. They collected data on types, number of, and size of ads (only those in English and Spanish). Data was coded by product content, and targeting of ads based on photographs. A fast food ad that featured two young men playing soccer would have been coded as both “fast food” and “physical activity”.	Minimum of 8 × 12 inches, virtually all billboards, bus shelter ads, bus bench ads, sidewalk sandwich signs, murals painted on the side of buildings, some store window posters. Not for a product or service specific to the premises on which the ad was located or the name of an establishment	Sugary beverages were featured in 4.1% of ads across all areas, fast food 2.6% and other food 8.2%. One ad promoted fresh fruit/veg. Salads were pictured on 8 (13%) of fast food ads in LA, fresh fruit and veg was shown in one ad for a grocery delivery service in New York. *Note: additional food data was not extracted due to being combined with alcoholic beverages*
Zenk et al. (2021) [68]	To evaluate long-term changes in store marketing practices two-years (24-months) post-tax implementation	Stores in Oakland compared to Sacramento, California, US	Repeated cross-sectional, observational, content analysis	Researchers completed in-store audits pre- and post-tax implementation. Price promotions (on sale or discounted price) were measured for SSBs, artificially sweetened beverages and unsweetened beverages.	Signs, posters, flags, decals, stickers, marquees and sandwich boards on the building exterior and property	31.4% of stores in Oakland had exterior SSB advertising (29% at supermarkets, 32.4% at limited service stores). Post-tax implementation, 24.5% of stores had exterior advertising (12.9% at supermarkets, 29.6% at limited service stores). Sacramento (no tax implemented) baseline = 37.3%, post-tax = 33.1% For exterior advertising, no significant changes were found in Oakland relative to Sacramento for SSBs, ASBs or USBs.

*Abbreviations*: *F&B* Food and beverage, *Ads* Advertisements, *SSBs* Sugar-sweetened beverages, *ASBs* Artificially sweetened beverages *USBs* Unsweetened beverages*, WHO* World Health Organization, *SES* socioeconomic status, *SEP* Socioeconomic position

Of studies including participants (*n* = 7), three measured exposure of children between the ages of 10 and 14 [89, 93, 102], two surveyed caregivers of children aged 3–5 [70] (79.2% mothers) and 0–2 years [85] (100% mothers) and two studies collected data from adults in select census tracts [53, 71].

#### What common criteria are used to define outdoor food marketing?

As shown in Tables 1, 2 and 3, the majority of studies (*n* = 33) encompassed a combination of outdoor media [53–55, 58, 60–62, 65–68, 72, 73, 75, 76, 78–80, 80–84, 87–89, 91, 92, 94, 97, 98, 102–104]. Many (*n* = 11) focused on advertising solely on public transport property [64, 70, 71, 86, 90, 93, 95, 99–101, 106], five studies focused exclusively on billboards [63, 74, 77, 85, 96] and four measured advertising outside stores or food outlets [56, 57, 59, 105].

**Fig. 2:**
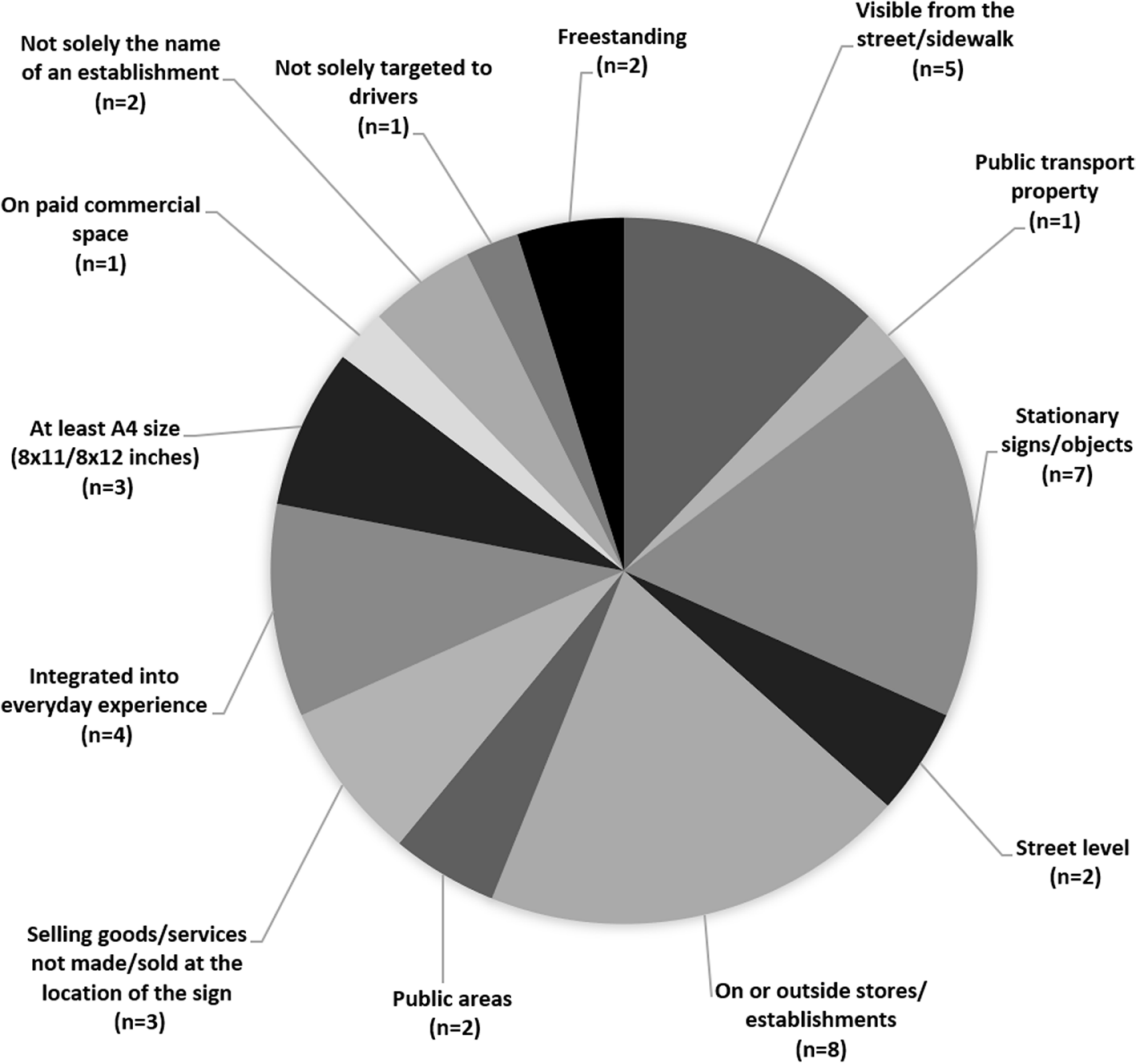
Common criteria used to define outdoor food marketing

Outdoor food marketing was inconsistently defined across studies. All studies stated the media they were measuring and some defined marketing or advertising generally, but often not how it related to the outdoor environment. Studies that provided specific criteria for outdoor food marketing (*n* = 15) or an equivalent term (i.e. outdoor food advertising) beyond simply stating the media recorded are listed in Table 4. Figure 2 represents the criteria referred to most frequently when defining outdoor food marketing.

**Table 4:** Outdoor food marketing definitions

Author(s), date, country	Definition of outdoor food (and beverage) marketing
**Adams et al. (2010), UK** [80]	All outdoor advertisements, for any product, within the study city boundaries.
**Adjoian et al. (2019), USA** [58]	Advertisements included in this study were street-level, stationary signs (posters, stickers, decals, etc.) that displayed a product with the intended purpose of promoting that product or type of product. One ad was considered the single, discrete, physical unit of the poster, sticker, decal etc., even if multiple products were featured.
**Barnes et al. (2016), USA** [59]	“Exterior marketing” (exterior of small stores) - Images on storefront doors or windows. Images defined to include brands, logos or texts of specified food items and well known products.
**Barquera et al. (2018), Mexico** [81]	Any [poster, banner, sticker, painting on walls, or flags] *inside* or outside stores and billboards and walls not related to stores in the buffer areas.
**Bragg et al. (2017) a, Ghana** [73]	Visible from the street (likely to allow for maximal consumer exposure - both pedestrians and drivers).
**Cassady et al. (2015), USA** [61]	Ads [found on billboards, bus shelters, bus benches, and posters on storefronts] large enough to be seen from the street.
**Chacon et al. (2015), Guatemala** [82]	[Posters, stickers, free-standing signs, banners, painting on walls, or flags Inside or] outside stores.
**Dia et al. (2021), Uganda** [75]	A sign with branded information, pictures or logos for food or beverage products or companies.
**Dowling et al. (2020), USA** [62]	Outdoor, street level stationary signs on outdoor structures, if they displayed a product with the intended purpose of promoting that product or type of product.
**Egli et al. (2019), New Zealand** [83]	Stationary objects containing either a recognisable logo and/or an intended message.
**Herrera & Pasch, (2018), USA** [55]	A marketing strategy that has the potential to influence the commercial food landscape.
**Huang et al. (2020), New Zealand** [86]	Stationary objects containing either a recognisable logo and/or an intended message.
**Isgor et al. (2016), USA** [56]	Advertisements posted on external sites such as billboards, wall signs, storefronts. Minimum of 8.5 × 11”.
**Kelly et al. (2008), Australia** [87]	[Billboards/posters with standard commercial advertisements, temporary advertising of special events, advertisements on outdoor furniture, and signs on buildings] with additional branded product information.
**Kelly et al. (2015), Mongolia, Philippines** [88]	Outdoor advertising in particular works by integrating branded messages into daily activities and the cultural landscape – signs with branded information, pictures or logos … signage with a product logo in additio n to store identification.
**Liu et al. (2020), New Zealand** [89]	In outdoor public areas.
**Lowery & Sloane (2014), USA** [63]	A category of signage that advertises goods or services that are not made or sold at the location of the sign.
**Maher et al. (2005), New Zealand** [91]	Stationary objects containing either a recognisable logo and/or an intended message.
**Nelson et al. (2020), Jamaica** [76]	On-premise business signage (“basic information about the products and services that are nearby” found on the outside of stores or restaurants). OOH: visible 24 hours a day and offers repeated exposures.
**Ohri-Vachaspati et al. (2015), USA** [57]	Posted on exterior of restaurants and visible from the parking lot or street.
**Palmer et al. 2021, UK** [94]	Prominent features of environments that individuals may experience and interact with in their everyday experiences.
**Pasch and Poulos (2013), USA** [65]	Free standing, located on something other than a building, or any advertisement directly attached to a building.
**Poulos & Pasch (2015), USA** [66]	Any sign promoting food or beverages that is free-standing or not attached to an establishment. AND any sign promoting food or beverages that is directly attached to or located on an establishment.
**Puspikawati et al. (2020), Indonesia** [97]	All food and beverage adverts that were visible from all the roads and alleys in the study location.
**Timmermans et al. (2018), Netherlands** [103]	Advertisements on stationary objects in public space.
**Trapp et al. (n.d.)** [104]	Stationary objects containing either a recognisable logo and/or an intended message.
**Vandevijvere et al. (2018), New Zealand** [78]	Signs of at least A4 size with branded information, pictures or logos.
**Velazquez et al. (2019), Canada** [79]	Posters or other physical materials with branded or non-branded information, images related to food, or logos for provincially or nationally recognizable food or beverage retailers with the intent to relay information and/or increase awareness about a particular food or beverage product.
**Watson et al. (2021), Australia** [106]	On public transport property.
**Yancey et al. (2009), USA** [67]	Advertising for a product or service visible from publicly accessible street or sidewalk, a minimum of 8 × 12 inches, posted on paid commercial space, not an ad for a product/service specific to premises on which the ad was located, not merely the name of the establishment, not targeted exclusively to drivers on high-speed thoroughfares, convey thematic content through words/pictures.
**Zenk et al. (2021), USA** [68]	On the building exterior and property.

#### What methods are used to document outdoor food marketing exposure, power, and impact?

Most included studies (*n* = 49) were cross-sectional, although four were longitudinal [68, 90, 95, 100]. The methods used to classify foods were inconsistent, for example, often local nutrient profiling models were used to classify advertised products as healthy or not healthy (e.g. [80]), however in some cases the number of advertisements for specific food groups were tallied (e.g. [56]). Forty-two studies assessed the frequency of food advertising through researcher visits to locations. In four [82, 83, 86, 93] studies, researchers visited streets virtually, through Google Street view. Real, rather than potential exposure was measured in three studies [89, 93, 102]. In two cases, children wore cameras which documented advertisements encountered in their typical day [89, 102], and in a final study, children wore a global positioning system (GPS) device so researchers could track when they encountered previously identified advertisements [93]. Self-reported retrospective exposure (frequency of encountering outdoor food advertising) was measured in three studies [70, 71, 85].

When measuring advertising around schools/places children gather, researchers typically created buffer zones, ranging from 100 m [81] to 2 km [105], with 500 m being the most frequent buffer size (*n* = 8) [77, 78, 86–88, 95, 97, 104]. Four studies used multiple buffers [82, 87, 93, 97], allowing for comparison between the area directly surrounding a school (e.g. < 250 m) with an area further away (e.g. 250-500 m) [87], one study compared advertising in Mass Transit Railway stations in school and non-school zones [90], and another used GPS point patterns to determine the extent of advertising around schools [92].

Content analysis was used to characterise the food types promoted and strategies used in the advertising. Two studies investigated price promotions [55, 56], two identified promotional characters and premium offers [75, 78], one specifically assessed child-directed marketing [57]. Others examined a mix of strategies including sports or health references, cultural relevance and emotional, value or taste appeals [54, 73, 74, 76, 79]. 

All three studies measuring the impact of outdoor food advertising used self-reported data. In a study conducted in Indonesia [70], caregivers reported the frequency of food advertising exposures in the past week, and their children’s frequency of intake of various confectionaries at home in the last week. In a study conducted in the US [53], individuals reported consumption of 12 oz. sodas in the last 24 hours, and odds of exposure was assessed by the extent of advertising in surrounding areas. In the UK study [71], participants reported exposure to HFSS advertising in the past week, and body mass index was calculated from participants’ reported height and weight.

#### What is known about exposure to outdoor food marketing?

##### Content of food marketing

Fifty-three studies investigated outdoor marketing exposure (Tables 1, 2 and 3), *n* = 22 reported specifically on exposure around schools or places children gather, *n* = 9 documented exposure on public transport and *n* = 4 outside stores/establishments. The remaining *n* = 21 measured exposure across multiple settings.

Food products were promoted in between 7.8% [64] and 57% [91] of advertisements, the mean across studies was 22.1%. Of food advertisements, a majority (~ 63%: range 39.3% [64] - 89.2% [89]) were categorised as unhealthy. Healthier foods were advertised far less, with studies generally reporting between 1.8% [97] and 18.8% [91] of food advertisements being for healthier products. Fast food (*n* = 17) and sugar-sweetened beverages (SSBs; *n* = 22) were frequently n amed as some of the most advertised product types.

Coca Cola was frequently stated as the most prominent brand advertised [73, 75, 88, 91, 100]. Around 5% of all outdoor food advertisements in New Zealand [78] and Australia [100] were promoting a brand (rather than a specific product), however there were no brand only advertisements identified in a UK study [80].

##### Marketing to children

Over half of the studies included (*n* = 29) sought to examine children’s exposure to food advertising. One UK study [93] concluded that while it was unlikely that unhealthy products were advertised on bus shelters surrounding schools (100-800 m), children, particularly in urban areas, were likely to encounter advertising on their journeys to and from school. All other studies found food advertising to be prevalent around schools, often promoting unhealthy products, although in three studies, a minority of schools (20.4% [78], 15.4% [79], 33.3% [103]) did not have any food advertising nearby. Four studies found that there was more food advertising closer to schools or facilities used by children and adolescents, compared to areas further away from these facilities [87, 97], specifically for unhealthy or processed foods [87, 90] and snack foods [82], however one study found SSB advertisements increased as distance from schools increased [92].

##### Differences by socioeconomic position/ethnicity

Eight studies considered differences in exposure by ethnicity. Three of these found that ethnic minority groups were exposed to more food advertising [55, 58, 62], for example, schools in the US with a majority Hispanic population were found to have more total advertisements and establishment advertisements in surrounding areas [55], whilst in New York City, for every 10% increase in proportion of Black residents there was a 6% increase in food images and 18% increase in non-alcoholic beverage images [58]. In addition to this, associations were found between sugary drink advertisement density and Percentage of Asian or Pacific Islander residents and percentage of Black, non-Latino residents [62]. Two studies found that multicultural neighbourhoods had a higher proportion of food advertisements [94] and higher density of unhealthy beverage advertisements [61]. Unhealthy food [63] and beverage [61] advertising were found to be more prevalent in ethnic minority communities. Low-income communities with majority Black or Latino residents had greater odds of having any food advertising [53], generally more food and beverage advertising and greater unhealthy food space [61] compared to white counterparts. A US study [56] found that differences in exposure to food and beverage, and soda advertisements by ethnicity were no longer significant after controlling for household income.

Twenty-six studies considered differences in exposure by SES, five of these did not find a relationship [60, 71, 83, 93, 99]. Two studies showed that food and beverage advertisements were more prevalent in low SES communities [56, 94]. Schools characterised by low SES had a higher proportion unhealthy food advertising nearby in two studies [78, 104], although in one instance there was no significant difference in the number of unhealthy food advertisements [78]. One study conducted in Sweden [84] found no significant difference in the proportion of food advertisements by SES, however there was a significantly greater proportion of advertisements promoting ultra-processed foods in the more deprived region.

Foods more frequently advertised in low SES areas were: SSBs, hamburgers and kebabs, diet soft drinks, vegetable snacks, dairy with no added sugar [103], staple foods [91], flavoured milk and fruit juice [101]. Low income communities in the US had lower odds of fruit and vegetable advertisements at limited service stores [56] and a higher density of unhealthy beverages [61, 62] compared with higher-income communities.

Two studies found no significant difference in the number of core advertisements by SES [98, 100], however a study of outdoor food advertising in Uganda found that there were more healthy food advertisements in high income areas [75]. Advertisements for fast food, takeaways, hot beverages and soft drinks were found to be more frequent in high-income areas [91, 96, 101].

A study comparing four schools of varying deprivation found the school with lowest deprivation had no advertisements but there was no clear trend in extent of advertising by deprivation [105]. Two studies conducted in Mexico [81] and New Zealand [86] found outdoor food advertising to be more frequent around public schools than private schools, however a study in Uganda [75] found no significant difference in the number of core foods advertised around private and government funded schools, in all three studies private school was considered a proxy of high SES. In this New Zealand study, low decile areas had the greatest number of advertisements for non-core food, core food and non-core food and beverage, however when high decile schools were combined with areas around private schools, the greatest number of all food and beverage advertisements and non-core advertisements were found in high SES areas [86].

#### What is known about the power of outdoor food marketing?

**Fig. 3:**
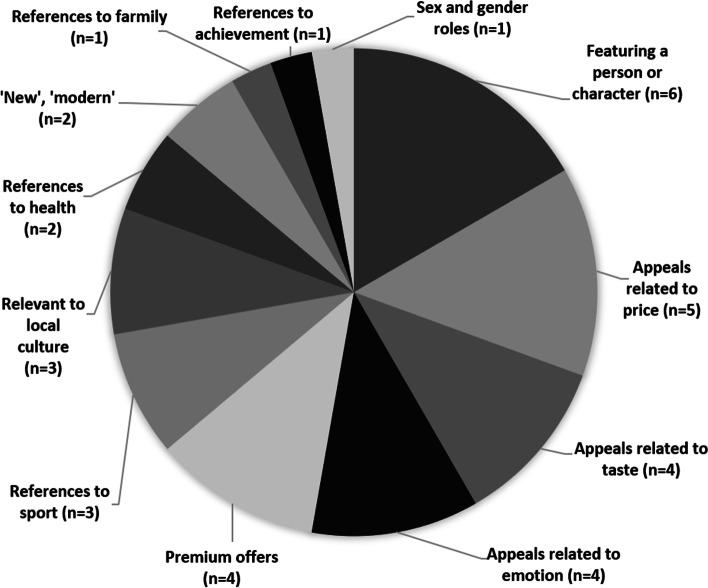
Powerful creative strategies observed in studies

Twelve studies documented the power of outdoor food marketing (Table 2). This was measured by quantifying the use of a range of persuasive creative strategies and child-directed marketing. The persuasive creative strategies observed across studies are shown in Fig. 3.

##### Observed power

There was evidence of variation in the use of persuasive creative strategies in outdoor advertising, with premium offers (e.g. buy one get one free [78]) utilised in between 7.84% [74] and 28.1% [78] of food advertisements, and the proportion of advertisements featuring a person or promotional character ranging from 2.8% [79] to 46.8% [73]. Other strategies frequently identified were appeals related to price [55, 56, 72, 74, 76], emotion [72, 74, 76, 77] and taste [72, 74, 76, 77].

The proportion of advertisements considered to be targeted just at children or young people ranged from less than 1% [58] to 10.4% [73]. Studies assessing appeals to children considered the use of cartoon characters, popular figures, child models or characters, colours or images, toys and the placement of the advertisement [58, 64, 73, 83].

Often, the foods promoted using persuasive creative strategies were soft drinks [73, 76], non-core foods [77] and fast foods [57, 76], however one study [75] observed outdoor food advertising in Uganda and found that 15% of healthy food advertisements used promotional characters.

##### Differences by socioeconomic status/ethnicity

A US study [55] found that schools with a majority Hispanic population (vs. low Hispanic population) had significantly more advertisements featuring price promotions within half a mile of the school. Price promotions were also more frequent outside supermarkets in non-Hispanic Black communities in the US [56], although this was no longer significant after controlling for household income. Supermarkets in low-income communities were significantly more likely to have price promotions [56] and being located in middle-income (compared to high) and black communities was marginally associated with increased odds of child-directed marketing [57]. Sometimes, local culture was referenced in food advertising through persuasive creative strategies [54, 76, 77], for example a US study quantifying advertisements in a Chinese-American Neighbourhood [54] found food advertisements were frequently relevant to Chinese culture (58.9% of food and 59.04% of non-alcoholic beverage advertisements), often featuring Asian models.

#### What is known about the impact of outdoor food marketing?

Three studies (Table 1) [53, 70, 71] explored associations between exposure to outdoor food advertising and behavioural or health outcomes, two of these found a significant positive relationship. Lesser et al. (2013) [53] found that for every 10% increase in outdoor food advertisements present, residents consumed on average 6% more soda, and had 5% higher odds of living with obesity. In Indonesia [70] self-reported exposure to food advertising on public transport was associated with consumption of two specific HFSS products. No associations were found between exposure and consumption of the other eight products considered. A UK study [71] found no significant association between self-reported exposure to HFSS advertising across transport networks and weight status. No studies measured differences in impact in relation to equity characteristics.

## Discussion

### Summary of main results

This review is the first to collate the criteria used to define outdoor food marketing, document the methods used to measure this form of marketing, and identify what is known about its exposure, power and impact.

Fifty-three studies were identified which met all eligibility criteria. In brief, of studies with a definition, the criteria referenced most were; on or outside stores/establishments; and stationary signs/objects. The methods used to research outdoor marketing include self-report data, virtual auditing, in-person auditing, and content analysis. There was little consistency in the approach used to classify foods as healthy or unhealthy, although nutrient profiling models were used in some studies.

Food accounted for an average of 22.1% of all advertisements, the majority of foods advertised were classed as unhealthy (63%). Ethnic minority groups were generally shown to have higher exposure to outdoor food advertising, but findings on differential exposure by SES were inconsistent.

Studies showed frequent use of premium offers, promotional characters, health claims, taste appeals and emotional appeals in outdoor food advertisements. There was limited evidence of relationships between exposure to food marketing and behavioural or health outcomes.

### Common criteria used to define outdoor food marketing

Eight out of fifteen studies (Fig. 2) stated that outdoor food marketing must be on or outside of stores or establishments, seven studies included stationary signs or objects in their definition and five studies stated that advertisements must be visible from the street or sidewalk. However, the defining criteria was inconsistent across the fifteen studies, and some of the most referenced criteria are problematic. Although stationary signage is an important aspect of outdoor marketing, this excludes forms of marketing on transport e.g. the exterior of buses. Equally, not all outdoor marketing may be “visible from the street or sidewalk”, this could exclude advertising on public transport property, i.e. station platforms. Additionally, the share of digital out-of-home advertising rose from 14% in 2011 to 59% in 2020 [107]. Three studies did aim to document digital advertising [60, 63, 90] through observing a digital board for a set amount of time. This medium is likely to become more prevalent over time globally, and there are challenges due to its changing and interactive nature [108]. The literature appears dominated by studies of advertising. This may reflect that most marketing encountered outdoors is advertising, conversely, it may be that the literature is yet to consider some newer forms of marketing, such as increased digital platforms. It will be important for future research to consider the evolving nature of outdoor marketing and how this should be measured.

Only fifteen studies defined outdoor food marketing as a term. This has likely been a factor influencing the heterogeneity observed across studies (e.g. differences in scope), as inconsistencies in defining a factor can negatively impact the development of an evidence base [109]. Researchers should endeavour to work towards an agreed definition, perhaps through use of the Delphi method of consensus development [110], in order to improve consistency in the resulting research. However, this method can be open to bias if the researchers are of the same background as the experts involved [109] therefore it is important that any definition developed aligns with criteria used by industry to reduce likelihood of bias.

### Methods used in outdoor food marketing research

Outdoor food marketing exposure and impact were measured using self-reported data, which may lack validity, as advertising can influence brand attitudes whether consciously or unconsciously processed [111]. While it can be useful to know the extent that individuals process advertising, this may not be a true representation of exposure. Equally, participants may alter their response to appear socially desirable which has previously resulted in misreporting of height and weight data [112].

Using Google Street View as an auditing tool is beneficial in saving time and resources whilst gathering large samples [113], however almost one third of advertisements in one study were unable to be identified [83], therefore systematically searching the streets in sample areas, and taking photographs for later reference is a more reliable method. Buffer areas are a useful tool for measuring advertising, particularly around specific sites such as schools, although stating advertising was present “around schools” has different meanings when comparing 100 m to 500 m, or to 2 km. GPS and wearable camera technology can identify how individuals encounter food marketing in the routes they use to travel through their environment. These methods should be replicated globally as a more objective measure of individual exposure to outdoor food marketing, although care must be taken in regard to privacy and ethical considerations.

There was little consistency in the methods used to identify persuasive creative strategies, which is typical in the field of food marketing [23]. The heterogeneity observed could be reduced through adherence to protocols for the monitoring of food marketing such as those developed by WHO [51] and INFORMAS [114]. This would improve comparability of future outdoor food marketing data across countries and time points which would better support policy action in this area. Nutrient profiling models are a useful tool for food categorisation, as opposed to grouping foods as “everyday” and “discretionary” or “core” and “non-core”, however, profiling models differ due to cultural differences in diet [106, 115]. There is a need to balance the data required for country-level policy relevance with international comparability. Watson et al. (2021) [106] propose an amalgamation of the WHO EURO NPM and WHO Western Pacific models.

### Exposure to outdoor food marketing

Marketing platforms outdoors remain accessible for the food industry and are relatively unrestricted. This is reflected by the extent of advertised food products (22.1%) and the proportion of those that were unhealthy (63%), which is problematic as discrepancies between the food types frequently promoted and dietary recommendations have been linked to changes in dietary norms and food preferences [111]. Whilst fruits and vegetables should make up 40% of daily intake [116], these products were rarely promoted. These findings are comparable to global data of other marketing formats, for example, a benchmarking study found that on average, 23% of advertisements on TV were for foods or beverages, and other studies have found 60–70% of food advertisements to be unhealthy across social media [117–119] and in print [120].

This knowledge adds to the existing evidence reporting the extent of children’s exposure through multiple forms of marketing [2, 13, 121]. Whilst efforts are being made to restrict their advertising exposure through other sources such as TV, for consistency, more must be done to protect children in the outdoor environment.

There is no consensus on clear trends in exposure by SES. In part, contradictory findings within this review, such as targeting of wealthier consumers, may reflect the occurrence of a nutrition transition occurring in low income countries, characterised by increased reliance on processed foods [122] which are more available to those with more disposable income. Further research should attempt to develop clear consensus on the differential exposure to outdoor food marketing by SES in both high- and lower-income countries.

### Power of outdoor food marketing

The lack of research into the power of outdoor food marketing is most likely a result of the lack of established definitions and classifications for the powerful characteristics of marketing and in particular, child appeal of marketing [123]. The most frequent persuasive creative strategies identified across the twelve studies documenting power were premium offers, promotional characters, health claims, taste appeals and emotional appeals, similar to those identified in television food marketing [23]. These strategies are particularly salient to children: spokes-characters can be effective in influencing children’s food choice, preference, awareness and attention [124], whilst premium offers (e.g. collectible toys) can influence children’s likeability and anticipated taste of the promoted food [125] and can prompt choice of healthier meals [125, 126]. One study found that children were more likely to choose unhealthy food products if they featured nutrient content claims such as “reduced fat, source of calcium” [127]. In this study participants were exposed to unknown brands, it is anticipated that larger responses would be present in brands recognised by participants. Future research should attempt to determine the success of different strategies in influencing behaviour, particularly as the rise in digital media used outdoors may increase the potential for power through increasing the variation and sophistication of outdoor marketing techniques. Policy in this field is largely focused on advertising directed at children, although it is important for research and policy to reflect that due to persuasive creative strategies used, advertising not wholly directed at children can still appeal to them [83].

### Impact of outdoor food marketing

There is evidence that outdoor advertising exposure is related to consumption of SSBs and odds of obesity, Previous reviews on the impact of food marketing on television and digital media have found compelling evidence of a relationship between exposure and food intake [44], attitudes and preferences [24]. However, the small number of studies measuring impacts of outdoor food marketing in this review were correlational and therefore cannot demonstrate causality. This lack of evidence is likely preventing policy progress in this area. It is likely that the lack of studies measuring impact of outdoor food marketing is due to the difficulty in controlling for confounding variables in external settings [128] or replicating this form of marketing in a lab compared to other formats such as television. It is clear that unhealthy food marketing is prevalent outdoors, but our understanding of the resultant impacts is underdeveloped and must be further examined through experimental research.

Experimental research will enable clearer understanding as to whether outdoor food marketing influences behaviour as television and digital marketing do [10, 24, 129]. Understanding the impact of outdoor food marketing on body weight would require longitudinal research, although it is difficult to separate the impact of marketing from secular trends. Additionally, there is increasing recognition that attributing a behavioural outcome to a single marketing communication can be problematic and does not appropriately reflect the cumulative effects of multiple, repeated exposures [7]. Purchase data in response to marketing campaigns could be a useful indicator of marketing impact [130], however gathering sales data from industry is problematic. This could be made possible through changes such as those proposed by the UK national food strategy, and supported by the NGO sector [131], calling for mandatory annual reporting of product sales for large food companies [132]. Although this is only proposed in the UK, if the strategy is successful in encouraging companies to make changes to formulations or the proportion of healthy products available, this strategy may be adopted elsewhere.

### Strengths

The review was pre-registered, allowing for transparency in approach and reporting of results and the methodology and reporting of the review were robust and consistent with guidelines from both the PRISMA extension for scoping reviews and the JBI methodology. The systematic search strategy ensured a wide range of databases were searched and the identification of a large number of potentially relevant studies. The use of multiple independent reviewers in the full-text screening and data extraction ensured all relevant data was captured accurately.

### Limitations

As this is a scoping review, non-peer reviewed sources such as letters to editors [96], conference abstracts [99] and grey literature [104] were included if they met inclusion criteria. Government websites beyond the UK were not included, which is a limitation of our searches, however multiple grey literature sources that were not UK based would have captured relevant international materials. The majority of studies included are focused on advertising and while some marketing aspects are considered, this review does not encompass all marketing communications. However, our searches were designed with thesaurus terms to capture words related to marketing that might not have been realised from a public health perspective. Therefore, it is likely that the relevant literature from marketing disciplines was identified, and is just limited.

As quality assessment was not deemed appropriate, there is potential for error and bias within the included studies, similarly, inaccuracies may arise from the self-reported data used in four studies. Although there were no limitations by language, no translation was required and all eligible studies were published in English. Further, discrepancies in the conduct and reporting of studies make it difficult to collate data and draw firm conclusions.

## Conclusions

This review has documented the research on outdoor food marketing exposure, power, and impact. There is substantial heterogeneity in the criteria used to define and methods used to measure outdoor food marketing. Future research will benefit from using a consistent definition and measurement tools to allow for improved comparability between studies. Whilst all the studies documented exposure, few recorded the powerful strategies used in outdoor food marketing and it is still largely unknown how this marketing influences behaviour and ultimately health. In order to inform policy, further research will benefit from examining the causal processes through which outdoor marketing may influence behaviour and health outcomes.

## Supplementary Information

**Additional file 1.**

## Data Availability

The datasets generated and/or analysed during the current study are available in the OSF repository, https://osf.io/b65jy/.

## Abbreviations

AMA: American Marketing Association
GPS: Global Positioning System
HFSS: High in fats, salt and sugar
INFORMAS: International Network for Food and Obesity/Non-communicable Diseases Research, Monitoring and Action Support.
NPM: Nutrient Profiling Model
PROGRESS: Place, race/ethnicity/culture/language, occupation, gender/sex, religion, education, socioeconomic status, social capital
SES: Socioeconomic status
SSBs: Sugar-sweetened beverages
WHO: World Health Organization
