# Supplementary material for: A scoping review of outdoor food marketing: exposure, power and impacts on eating behaviour and health
Source: BMC Public Health. 2022 Jul 27;22:1431. doi: 10.1186/s12889-022-13784-8 (PMC9330687; doi:10.1186/s12889-022-13784-8)
Supplement: Supplementary file 1 — Additional file 1. [file 12889_2022_13784_MOESM1_ESM.docx]

**Supplementary material 1: Search strategy**

Free-text and thesaurus terms of MEDLINE records of key studies were used, and the search strategy amended to ensure it captured all relevant records. The final search strategy was revised for each database/information source, all including food and beverage, marketing, and outdoor terms. All databases were searched from inception and there were no restrictions by year or language.

For Grey literature sources, simple searches (“outdoor food marketing”, “outdoor food advertising” were undertaken of publications and reports. An initial screening of titles was undertaken by one researcher, and any sources deemed possibly relevant were put through to the full-text screening where they were screened by the primary author and one other.

Searches were conducted between January 21, 2021 and February 10, 2021. Forward citation searches of included studies were completed on May 01, 2021 to identify any recent research that had been conducted.

**Medline (ovid) search strategy - 759**

| 1. exp Food/ (1308900) |  |
| --- | --- |
| 2. exp Food Industry/ (185450) |  |
| 3. beverages/ or artificially sweetened beverages/ or exp carbonated beverages/ or coffee/ or exp drinking water/ or energy drinks/ or "fruit and vegetable juices"/ or exp milk/ or exp milk substitutes/ or sugar-sweetened beverages/ or exp tea/ or teas, herbal/ or teas, medicinal/ (129961) |  |
| 4. food.ti,ab,kw. (441659) |  |
| 5. foods.ti,ab,kw. (86257) |  |
| 6. drink.ti,ab,kw. (20865) |  |
| 7. drinks.ti,ab,kw. (16304) |  |
| 8. HFSS.ti,ab,kw. (215) |  |
| 9. sugar-sweetened.ti,ab,kw. (3177) |  |
| 10. sugar.ti,ab,kw. (94330) |  |
| 11. high-sugar.ti,ab,kw. (2185) |  |
| 12. fat.ti,ab,kw. (271721) |  |
| 13. salt.ti,ab,kw. (146719) |  |
| 14. ((carbonated or fizzy or non-alcoholic) adj beverage*).ti,ab,kw. (879) |  |
| 15. snack*.ti,ab,kw. (8361) |  |
| 16. junk-food.ti,ab,kw. (609) |  |
| 17. fast-food.ti,ab,kw. (3310) |  |
| 18. take-away*.ti,ab,kw. (451) |  |
| 19. 1 or 2 or 3 or 4 or 5 or 6 or 7 or 8 or 9 or 10 or 11 or 12 or 13 or 14 or 15 or 16 or 17 or 18 (2102426) |  |
| 20. ((outdoor* or outside or out-of-home or OOH or DOOH or "area around" or street* or highway* or station or stations or subway or bus or buses or train or trains or taxi* or airport* or transport or transit or signage or telephone booth* or telephone kiosk* or billboard* or poster or posters or public space* or public place* or exterior*) adj10 (advert* or market* or media)).ti,ab,kw. (5203) |  |
| 21. exp Marketing/ and (outdoor* or outside or out-of-home or OOH or DOOH or "area around" or street* or highway* or station or stations or subway or bus or buses or train or trains or taxi* or airport* or transport or transit or signage or telephone booth* or telephone kiosk* or billboard* or poster or posters or public space* or public place* or exterior*).ti,ab,kw. (1182) |  |
| 22. 20 or 21 (5943) |  |
| 23. 19 and 22 (908) |  |
| 24. exp animals/ (23770725) |  |
| 25. human/ (18988986) |  |
| 26. 24 not 25 (4781739) |  |
| 27. 23 not 26 (759) |  |

**Cochrane Library - 273**

ID Search

#1 MeSH descriptor: [Food] explode all trees

#2 MeSH descriptor: [Food Industry] explode all trees

#3 MeSH descriptor: [Beverages] this term only

#4 MeSH descriptor: [Artificially Sweetened Beverages] explode all trees

#5 MeSH descriptor: [Carbonated Beverages] explode all trees

#6 MeSH descriptor: [Coffee] explode all trees

#7 MeSH descriptor: [Drinking Water] explode all trees

#8 MeSH descriptor: [Energy Drinks] explode all trees

#9 MeSH descriptor: [Fruit and Vegetable Juices] explode all trees

#10 MeSH descriptor: [Milk] explode all trees

#11 MeSH descriptor: [Milk Substitutes] explode all trees

#12 MeSH descriptor: [Sugar-Sweetened Beverages] explode all trees

#13 MeSH descriptor: [Tea] explode all trees

#14 MeSH descriptor: [Teas, Herbal] explode all trees

#15 MeSH descriptor: [Teas, Medicinal] explode all trees

#16 food:ti,ab,kw

#17 foods:ti,ab,kw

#18 drink:ti,ab,kw

#19 drinks:ti,ab,kw

#20 HFSS:ti,ab,kw

#21 sugar-sweetened:ti,ab,kw

#22 sugar:ti,ab,kw

#23 high-sugar:ti,ab,kw

#24 fat:ti,ab,kw

#25 salt:ti,ab,kw

#26 ((carbonated or fizzy or non-alcoholic) NEXT beverage*):ti,ab,kw

#27 snack:ti,ab,kw

#28 junk-food:ti,ab,kw

#29 fast-food:ti,ab,kw

#30 take-away:ti,ab,kw

#31 {OR #1-#30}

#32 ((outdoor* or outside or out-of-home or OOH or DOOH or "area around" or street* or highway* or station or stations or subway or bus or buses or train or trains or taxi* or airport* or transport or transit or signage or telephone booth* or telephone kiosk* or billboard* or poster or posters or public space* or public place* or exterior*) NEAR/10 (advert* or market* or media)):ti,ab,kw

#33 MeSH descriptor: [Marketing] explode all trees

#34 (outdoor* or outside or out-of-home or OOH or DOOH or "area around" or street* or highway* or station or stations or subway or bus or buses or train or trains or taxi* or airport* or transport or transit or signage or telephone booth* or telephone kiosk* or billboard* or poster or posters or public space* or public place* or exterior*):ti,ab,kw

#35 #33 AND #34

#36 #32 OR #35

#37 #31 AND #36

**CINAHL – 405**

S1 (MH "Food+")

S2 (MH "Food Industry+")

S3 (MH "Beverages") OR (MH "Carbonated Beverages") OR (MH "Coffee") OR (MH "Energy Drinks") OR (MH "Fruit Juices+") OR (MH "Kombucha") OR (MH "Milk") OR (MH "Milk Substitutes+") OR (MH "Sports Drinks") OR (MH "Sweetened Beverages") OR (MH "Tea") OR (MH "Water+")

S4 TI food OR AB food OR SU food

S5 TI foods OR AB foods OR SU foods

S6 TI drink OR AB drink OR SU drink

S7 TI drinks OR AB drinks OR SU drinks

S8 TI HFSS OR AB HFSS OR SU HFSS

S9 TI sugar-sweetened OR AB sugar-sweetened OR SU sugar-sweetened

S10 TI sugar OR AB sugar OR SU sugar

S11 TI high-sugar OR AB high-sugar OR SU high-sugar

S12 TI fat OR AB fat OR SU fat

S13 TI salt OR AB salt OR SU salt

S14 TI ( ((carbonated or fizzy or non-alcoholic) N0 beverage*) ) OR AB ( ((carbonated or fizzy or non-alcoholic) N0 beverage*) ) OR SU ( ((carbonated or fizzy or non-alcoholic) N0 beverage*) )

S15 TI snack* OR AB snack* OR SU snack*

S16 TI junk-food OR AB junk-food OR SU junk-food

S17 TI fast-food OR AB fast-food OR SU fast-food

S18 TI take-away OR AB take-away OR SU take-away

S19 S1 OR S2 OR S3 OR S4 OR S5 OR S6 OR S7 OR S8 OR S9 OR S10 OR S11 OR S12 OR S13 OR S14 OR S15 OR S16 OR S17 OR S18

S20 TI ( ((outdoor* or outside or out-of-home or OOH or DOOH or "area around" or street* or highway* or station or stations or subway or bus or buses or train or trains or taxi* or airport* or transport or transit or signage or telephone booth* or telephone kiosk* or billboard* or poster or posters or public space* or public place* or exterior*) N10 (advert* or market* or media)) ) OR AB ( ((outdoor* or outside or out-of-home or OOH or DOOH or "area around" or street* or highway* or station or stations or subway or bus or buses or train or trains or taxi* or airport* or transport or transit or signage or telephone booth* or telephone kiosk* or billboard* or poster or posters or public space* or public place* or exterior*) N10 (advert* or market* or media)) ) OR SU ( ((outdoor* or outside or out-of-home or OOH or DOOH or "area around" or street* or highway* or station or stations or subway or bus or buses or train or trains or taxi* or airport* or transport or transit or signage or telephone booth* or telephone kiosk* or billboard* or poster or posters or public space* or public place* or exterior*) N10 (advert* or market* or media)) )

S21 (MH "Marketing+")

S22 TI ( (outdoor* or outside or out-of-home or OOH or DOOH or "area around" or street* or highway* or station or stations or subway or bus or buses or train or trains or taxi* or airport* or transport or transit or signage or telephone booth* or telephone kiosk* or billboard* or poster or posters or public space* or public place* or exterior*) ) OR AB ( (outdoor* or outside or out-of-home or OOH or DOOH or "area around" or street* or highway* or station or stations or subway or bus or buses or train or trains or taxi* or airport* or transport or transit or signage or telephone booth* or telephone kiosk* or billboard* or poster or posters or public space* or public place* or exterior*) ) OR SU ( (outdoor* or outside or out-of-home or OOH or DOOH or "area around" or street* or highway* or station or stations or subway or bus or buses or train or trains or taxi* or airport* or transport or transit or signage or telephone booth* or telephone kiosk* or billboard* or poster or posters or public space* or public place* or exterior*) )

S23 S21 AND S22

S24 S20 OR S23

S25 S19 AND S24

**Proquest - 416**

S1 MESH.EXACT.EXPLODE("Food:J.02.500") OR MESH.EXACT.EXPLODE("Food:G.07.203.300")

1,402,177

S2 MESH.EXACT.EXPLODE("Food Industry")

189,808

S3 MESH.EXACT("Energy Drinks") OR MESH.EXACT("Fruit and Vegetable Juices") OR MESH.EXACT.EXPLODE("Artificially Sweetened Beverages:J.02.200.200") OR MESH.EXACT.EXPLODE("Drinking Water:J.02.200.418") OR MESH.EXACT.EXPLODE("Tea:J.02.200.831") OR MESH.EXACT("Teas, Medicinal") OR MESH.EXACT("Teas, Herbal") OR MESH.EXACT("Sugar-Sweetened Beverages") OR MESH.EXACT.EXPLODE("Milk Substitutes:J.02.200.712") OR MESH.EXACT.EXPLODE("Milk:J.02.200.700") OR MESH.EXACT("Coffee") OR MESH.EXACT("Beverages") OR MESH.EXACT.EXPLODE("Carbonated Beverages:J.02.200.300")

131,069

S4 noft(food) OR noft(foods) OR noft(drink) OR noft(drinks) OR noft(HFSS) OR noft(sugar-sweetened) OR noft(sugar) OR noft(high-sugar) OR noft(fat) OR noft(salt)

4,248,882

S5 noft(((carbonated or fizzy or non-alcoholic) N/ beverage*)) OR noft(snack*) OR noft(junk-food) OR noft(fast-food) OR noft(take-away)

84,107

S6 (MESH.EXACT.EXPLODE("Food:J.02.500") OR MESH.EXACT.EXPLODE("Food:G.07.203.300")) OR MESH.EXACT.EXPLODE("Food Industry") OR (MESH.EXACT("Energy Drinks") OR MESH.EXACT("Fruit and Vegetable Juices") OR MESH.EXACT.EXPLODE("Artificially Sweetened Beverages:J.02.200.200") OR MESH.EXACT.EXPLODE("Drinking Water:J.02.200.418") OR MESH.EXACT.EXPLODE("Tea:J.02.200.831") OR MESH.EXACT("Teas, Medicinal") OR MESH.EXACT("Teas, Herbal") OR MESH.EXACT("Sugar-Sweetened Beverages") OR MESH.EXACT.EXPLODE("Milk Substitutes:J.02.200.712") OR MESH.EXACT.EXPLODE("Milk:J.02.200.700") OR MESH.EXACT("Coffee") OR MESH.EXACT("Beverages") OR MESH.EXACT.EXPLODE("Carbonated Beverages:J.02.200.300")) OR (noft(food) OR noft(foods) OR noft(drink) OR noft(drinks) OR noft(HFSS) OR noft(sugar-sweetened) OR noft(sugar) OR noft(high-sugar) OR noft(fat) OR noft(salt)) OR (noft(((carbonated OR fizzy OR non-alcoholic) N/ beverage*)) OR noft(snack*) OR noft(junk-food) OR noft(fast-food) OR noft(take-away))

5,270,924

S8 noft(((outdoor* or outside or out-of-home or OOH or DOOH or "area around" or street* or highway* or station or stations or subway or bus or buses or train or trains or taxi* or airport* or transport or transit or signage or telephone booth* or telephone kiosk* or billboard* or poster or posters or public space* or public place* or exterior*) N10 (advert* or market* or media)))

24

S9 MESH.EXACT.EXPLODE("Marketing") AND noft(outdoor* or outside or out-of-home or OOH or DOOH or "area around" or street* or highway* or station or stations or subway or bus or buses or train or trains or taxi* or airport* or transport or transit or signage or telephone booth* or telephone kiosk* or billboard* or poster or posters or public space* or public place* or exterior*)

1,898

S10 noft(((outdoor* OR outside OR out-of-home OR OOH OR DOOH OR "area around" OR street* OR highway* OR station OR stations OR subway OR bus OR buses OR train OR trains OR taxi* OR airport* OR transport OR transit OR signage OR telephone booth* OR telephone kiosk* OR billboard* OR poster OR posters OR public space* OR public place* OR exterior*) N10 (advert* OR market* OR media))) OR (MESH.EXACT.EXPLODE("Marketing") AND noft(outdoor* OR outside OR out-of-home OR OOH OR DOOH OR "area around" OR street* OR highway* OR station OR stations OR subway OR bus OR buses OR train OR trains OR taxi* OR airport* OR transport OR transit OR signage OR telephone booth* OR telephone kiosk* OR billboard* OR poster OR posters OR public space* OR public place* OR exterior*))

1,922

S11 ((MESH.EXACT.EXPLODE("Food:J.02.500") OR MESH.EXACT.EXPLODE("Food:G.07.203.300")) OR MESH.EXACT.EXPLODE("Food Industry") OR (MESH.EXACT("Energy Drinks") OR MESH.EXACT("Fruit and Vegetable Juices") OR MESH.EXACT.EXPLODE("Artificially Sweetened Beverages:J.02.200.200") OR MESH.EXACT.EXPLODE("Drinking Water:J.02.200.418") OR MESH.EXACT.EXPLODE("Tea:J.02.200.831") OR MESH.EXACT("Teas, Medicinal") OR MESH.EXACT("Teas, Herbal") OR MESH.EXACT("Sugar-Sweetened Beverages") OR MESH.EXACT.EXPLODE("Milk Substitutes:J.02.200.712") OR MESH.EXACT.EXPLODE("Milk:J.02.200.700") OR MESH.EXACT("Coffee") OR MESH.EXACT("Beverages") OR MESH.EXACT.EXPLODE("Carbonated Beverages:J.02.200.300")) OR (noft(food) OR noft(foods) OR noft(drink) OR noft(drinks) OR noft(HFSS) OR noft(sugar-sweetened) OR noft(sugar) OR noft(high-sugar) OR noft(fat) OR noft(salt)) OR (noft(((carbonated OR fizzy OR non-alcoholic) N/ beverage*)) OR noft(snack*) OR noft(junk-food) OR noft(fast-food) OR noft(take-away))) AND (noft(((outdoor* OR outside OR out-of-home OR OOH OR DOOH OR "area around" OR street* OR highway* OR station OR stations OR subway OR bus OR buses OR train OR trains OR taxi* OR airport* OR transport OR transit OR signage OR telephone booth* OR telephone kiosk* OR billboard* OR poster OR posters OR public space* OR public place* OR exterior*) N10 (advert* OR market* OR media))) OR (MESH.EXACT.EXPLODE("Marketing") AND noft(outdoor* OR outside OR out-of-home OR OOH OR DOOH OR "area around" OR street* OR highway* OR station OR stations OR subway OR bus OR buses OR train OR trains OR taxi* OR airport* OR transport OR transit OR signage OR telephone booth* OR telephone kiosk* OR billboard* OR poster OR posters OR public space* OR public place* OR exterior*)))

416

**PsycINFO – 271**

S1 DE "Food" OR DE "Fast Food" OR DE "Food Additives" OR DE "Food Insecurity" OR DE "Food Preparation" OR DE "Food Safety"

S2 DE "Beverages (Nonalcoholic)" OR DE "Energy Drink"

S3 TI food OR AB food OR KW food

S4 TI foods OR AB foods OR KW foods

S5 TI drink OR AB drink OR KW drink

S6 TI drinks OR AB drinks OR KW drinks

S7 TI HFSS OR AB HFSS OR KW HFSS

S8 TI sugar-sweetened OR AB sugar-sweetened OR KW sugar-sweetened

S9 TI sugar OR AB sugar OR KW sugar

S10 TI high-sugar OR AB high-sugar OR KW high-sugar

S11 TI fat OR AB fat OR KW fat

S12 TI salt OR AB salt OR KW salt

S13 TI ( ((carbonated or fizzy or non-alcoholic) n0 beverage*) ) OR AB ( ((carbonated or fizzy or non-alcoholic) n0 beverage*) ) OR KW ( ((carbonated or fizzy or non-alcoholic) n0 beverage*) )

S14 TI snack* OR AB snack* OR KW snack*

S15 TI junk-food OR AB junk-food OR KW junk-food

S16 TI fast-food OR AB fast-food OR KW fast-food

S17 TI take-away OR AB take-away OR KW take-away

S18 S1 OR S2 OR S3 OR S4 OR S5 OR S6 OR S7 OR S8 OR S9 OR S10 OR S11 OR S12 OR S13 OR S14 OR S15 OR S16 OR S17

S19 TI ( ((outdoor* or outside or out-of-home or OOH or DOOH or "area around" or street* or highway* or station or stations or subway or bus or buses or train or trains or taxi* or airport* or transport or transit or signage or telephone booth* or telephone kiosk* or billboard* or poster or posters or public space* or public place* or exterior*) N10 (advert* or market* or media)) ) OR AB ( ((outdoor* or outside or out-of-home or OOH or DOOH or "area around" or street* or highway* or station or stations or subway or bus or buses or train or trains or taxi* or airport* or transport or transit or signage or telephone booth* or telephone kiosk* or billboard* or poster or posters or public space* or public place* or exterior*) N10 (advert* or market* or media)) ) OR KW ( ((outdoor* or outside or out-of-home or OOH or DOOH or "area around" or street* or highway* or station or stations or subway or bus or buses or train or trains or taxi* or airport* or transport or transit or signage or telephone booth* or telephone kiosk* or billboard* or poster or posters or public space* or public place* or exterior*) N10 (advert* or market* or media)) )

S20 DE "Marketing" OR DE "Digital Marketing" OR DE "Retailing" OR DE "Social Marketing"

S21 TI ( outdoor* or outside or out-of-home or OOH or DOOH or "area around" or street* or highway* or station or stations or subway or bus or buses or train or trains or taxi* or airport* or transport or transit or signage or telephone booth* or telephone kiosk* or billboard* or poster or posters or public space* or public place* or exterior* ) OR AB ( outdoor* or outside or out-of-home or OOH or DOOH or "area around" or street* or highway* or station or stations or subway or bus or buses or train or trains or taxi* or airport* or transport or transit or signage or telephone booth* or telephone kiosk* or billboard* or poster or posters or public space* or public place* or exterior* ) OR KW ( outdoor* or outside or out-of-home or OOH or DOOH or "area around" or street* or highway* or station or stations or subway or bus or buses or train or trains or taxi* or airport* or transport or transit or signage or telephone booth* or telephone kiosk* or billboard* or poster or posters or public space* or public place* or exterior* )

S22 S20 AND S21

S23 S19 OR S22

S24 S18 AND S23

**Scopus - 1426**

( TITLE-ABS-KEY ( food OR foods OR drink OR drinks OR hfss OR sugar-sweetened OR sugar OR high-sugar OR fat OR salt OR "carbonated beverage*" OR "fizzy beverage" OR "non-alcoholic beverage*" OR snack* OR junk-food OR fast-food OR take-away* ) AND TITLE-ABS-KEY ( ( ( outdoor* OR outside OR out-of-home OR ooh OR dooh OR "area around" OR street* OR highway* OR station OR stations OR subway OR bus OR buses OR airport* OR train OR trains OR taxi* OR transport OR transit OR signage OR "telephone booth*" OR "telephone kiosk*" OR billboard* OR poster OR posters OR "public place*" OR "public space*" OR exterior* ) W/2 ( advert* OR market* OR media ) ) ) AND NOT TITLE-ABS-KEY ( "alcoholic beverage" )

**Science direct - 8**

Outdoor and food and advertising (5)
Outdoor and food and marketing (6)
without duplicates – 8

**PubMed - 618**

#1 "food"[MeSH Terms]

631,684

#2 "food industry"[MeSH Terms]

185,878

#3 ("beverages"[MeSH Terms]) NOT ("alcoholic beverages"[MeSH Terms])

121,301

#4 food[Title/Abstract]

450,052

#5 foods[Title/Abstract]

86,563

#6 drink[Title/Abstract]

20,956

#7 drinks[Title/Abstract]

16,326

#8 HFSS[Title/Abstract]

223

#9 sugar-sweetened[Title/Abstract]

3,166

#10 sugar[Title/Abstract]

97,857

#11 high-sugar[Title/Abstract]

2,181

#12 fat[Title/Abstract]

272,713

#13 salt[Title/Abstract]

148,099

#14 "Carbonated beverage*"[Title/Abstract]

567

#15 "non-alcoholic beverage*"[Title/Abstract]

372

#16 snack*[Title/Abstract]

8,370

#17 junk-food[Title/Abstract]

606

#18 fast-food[Title/Abstract]

3,291

#19 take-away*[Title/Abstract]

451

#20 #1 OR #2 OR #3 OR #4 OR #5 OR #6 OR #7 OR #8 OR #9 OR #10 OR #11 OR #12 OR #13 OR #14 OR #15 OR #16 OR #17 OR #18 OR #19

1,482,018

#21 (outdoor*[Title/Abstract] OR outside[Title/Abstract] OR out-of-home[Title/Abstract] OR OOH[Title/Abstract] OR DOOH[Title/Abstract] OR "area around"[Title/Abstract] OR street*[Title/Abstract] OR highway*[Title/Abstract] OR station[Title/Abstract] OR stations[Title/Abstract] OR subway[Title/Abstract] OR bus[Title/Abstract] OR buses[Title/Abstract] OR train[Title/Abstract] OR trains[Title/Abstract] OR taxi*[Title/Abstract] OR airport*[Title/Abstract] OR transport[Title/Abstract] OR transit[Title/Abstract] OR signage[Title/Abstract] OR telephone booth*[Title/Abstract] OR telephone kiosk*[Title/Abstract] OR billboard*[Title/Abstract] OR poster[Title/Abstract] OR posters[Title/Abstract] OR public space*[Title/Abstract] OR public place*[Title/Abstract] OR exterior*[Title/Abstract]) N10 (advert* OR market* OR media)

3

#22 (outdoor*[Title/Abstract] OR outside[Title/Abstract] OR out-of-home[Title/Abstract] OR OOH[Title/Abstract] OR DOOH[Title/Abstract] OR "area around"[Title/Abstract] OR street*[Title/Abstract] OR highway*[Title/Abstract] OR station[Title/Abstract] OR stations[Title/Abstract] OR subway[Title/Abstract] OR bus[Title/Abstract] OR buses[Title/Abstract] OR train[Title/Abstract] OR trains[Title/Abstract] OR taxi*[Title/Abstract] OR airport*[Title/Abstract] OR transport[Title/Abstract] OR transit[Title/Abstract] OR signage[Title/Abstract] OR telephone booth*[Title/Abstract] OR telephone kiosk*[Title/Abstract] OR billboard*[Title/Abstract] OR poster[Title/Abstract] OR posters[Title/Abstract] OR public space*[Title/Abstract] OR public place*[Title/Abstract] OR exterior*[Title/Abstract]) AND (Marketing[Title/Abstract])

1,241

#23 #21 OR #22

1,244

#24 #20 AND #23

618

**Supplementary material 2: Data extraction instrument**

| **Data to be extracted** | **Elaboration** |
| --- | --- |
| Authors, year, country |  |
| Study design |  |
| Study objectives |  |
| Definition of outdoor marketing and mediums included | Or equivalent term used |
| Advertising mediums included | e.g. billboard, poster, bus shelter |
| Sample size | Participants included in final study  (if there are participants) |
| Participant characteristics | Age, group characteristics (e.g. parents) |
| Study setting | e.g. a school, city centre |
| Length of exposure to marketing | (if participants are exposed) |
| Methods | Brief outline |
| Outcome measures | Exposure/Power/Impact |
| Reported outcomes | Key outcomes, numerical data where possible |
| Consideration of PROGRESS+ and study outcomes relevant to these inequalities | Whether researchers are considering PROGRESS inequalities in their research (Place, race/ethnicity/culture/language, occupation, gender/sex, religion, education, socioeconomic status, social capital)  Comparing groups in terms of PROGRESS+ on effect of marketing on outcome measures. |
| Outcomes relevant to demographics | Comparing groups in terms of population level demographics (e.g. age and gender) on effect of marketing on outcome measures. |
| Funding source |  |
| Reported conflict of interest |  |

**Supplementary material 3: Sources excluded following full-text review**

| **Reference** | **Reason for exclusion** |
| --- | --- |
| Abachizadeh K, Ostovar A, Pariani A, Raeisi A. Banning Advertising Unhealthy Products and Services in Iran: A One-Decade Experience. Risk Management and Healthcare Policy. 2020;13:965. | Design – opinion paper |
| Adeigbe RT, Baldwin S, Gallion K, Grier S, Ramirez AG. Food and beverage marketing to Latinos: a systematic literature review. Health Education & Behavior. 2015 Oct;42(5):569-82. | Design - review |
| Backholer K, Gupta A, Zorbas C, Bennett R, Huse O, Chung A, Isaacs A, Golds G, Kelly B, Peeters A. Differential exposure to, and potential impact of, unhealthy advertising to children by socio‐economic and ethnic groups: A systematic review of the evidence. Obesity Reviews. 2021 Mar;22(3):e13144. | Design - review |
| Barragan NC, Noller AJ, Robles B, Gase LN, Leighs MS, Bogert S, Simon PA, Kuo T. The “sugar pack” health marketing campaign in Los Angeles County, 2011-2012. Health promotion practice. 2014 Mar;15(2):208-16. | Intervention – not food and drink marketing (health campaign) |
| Beaudoin CE, Fernandez C, Wall JL, Farley TA. Promoting healthy eating and physical activity: short-term effects of a mass media campaign. American journal of preventive medicine. 2007 Mar 1;32(3):217-23. | Intervention – outdoor not isolated |
| Boles M, Adams A, Gredler A, Manhas S. Ability of a mass media campaign to influence knowledge, attitudes, and behaviors about sugary drinks and obesity. Preventive Medicine. 2014 Oct 1;67:S40-5. | Intervention – outdoor not isolated |
| Bowman DD, Minaker LM, Simpson BJ, Gilliland JA. Development of a teen-informed coding tool to measure the power of food advertisements. International journal of environmental research and public health. 2019 Jan;16(21):4258. | Design - qualitative |
| Caldwell JI, Robles B, Tyree R, Fraser RW, Dumke KA, Kuo T. Does Exposure to the Choose Water Campaign Increase Parental Intentions to Promote More Water and Less Sugar-Sweetened Beverage Consumption?. American Journal of Health Promotion. 2020 Jun;34(5):555-8. | Intervention – outdoor not isolated |
| Caldwell JI, Robles B, Tyree R, Fraser RW, Dumke KA, Kuo T. Does Exposure to the Choose Water Campaign Increase Parental Intentions to Promote More Water and Less Sugar-Sweetened Beverage Consumption?. American Journal of Health Promotion. 2020 Jun;34(5):555-8. | Duplicate |
| Cervi MM, Agurs-Collins T, Dwyer LA, Thai CL, Moser RP, Nebeling LC. Susceptibility to food advertisements and sugar-sweetened beverage intake in non-Hispanic black and non-Hispanic white adolescents. Journal of community health. 2017 Aug;42(4):748-56. | Intervention – outdoor not isolated |
| Chemas-Velez MM, Gómez LF, Velasquez A, Mora-Plazas M, Parra DC. Scoping review of studies on food marketing in Latin America: Summary of existing evidence and research gaps. Revista de saude publica. 2020 Jan 10;53:107. | Design - review |
| Choi H, Reid LN. Promoting healthy menu choices in fast food restaurant advertising: Influence of perceived Brand healthiness, Brand commitment, and health Consciousness. Journal of health communication. 2018 Apr 3;23(4):387-98. | Intervention – no outdoor marketing |
| Cotto-Rivera E, Anthony D, Akin J, Bhargava V, Dekle M, Childers A, Lee JS. P137 Evaluation of Social Marketing Campaign to Improve Fruit and Vegetable Intake in SNAP-Ed Eligible Adult Georgians, 2017-2019. Journal of Nutrition Education and Behavior. 2020 Jul 1;52(7):S81. | Intervention – outdoor not isolated |
| Flint SW, McKenna J. Public transport and the promotion of unhealthy food and drink. The Lancet Public Health. 2018 Jul 1;3(7):e312. | Design – opinion paper |
| Fox TA, Berry B, Bielicki AK, DeLyser J, Pivonka E. Increasing Fruit and Vegetable Intake Through Innovative Marketing. Nutrition Today. 2003 Jan 1;38(1):21-4. | Design – opinion paper |
| Gase LN, Barragan NC, Robles B, Leighs M, Kuo T. A mixed-methods evaluation of the choose less, weigh less portion size health marketing campaign in Los Angeles County. American Journal of Health Promotion. 2015 Jul;29(6):e214-24. | Intervention – outdoor not isolated |
| Gelormini M, Damasceno A, Lopes SA, Maló S, Chongole C, Muholove P, Casal S, Pinho O, Moreira P, Padrão P, Lunet N. Street food environment in Maputo (STOOD Map): a cross-sectional study in Mozambique. JMIR research protocols. 2015;4(3):e98. | Design – study protocol (no data) |
| George KS, Roberts CB, Beasley S, Fox M, Rashied-Henry K, Brooklyn Partnership to Drive Down Diabetes (BP3D). Our health is in our hands: a social marketing campaign to combat obesity and diabetes. American Journal of Health Promotion. 2016 Mar;30(4):283-6. | Intervention – not food and drink marketing |
| Gray H, Lovett S, Berumen J. P89 Grocery Store Observations Using the CX3 Tool in Underserved Neighborhoods in Tampa, FL. Journal of Nutrition Education and Behavior. 2020 Jul 1;52(7):S58. | Intervention – outdoor not isolated |
| Grier SA, Kumanyika SK. The context for choice: health implications of targeted food and beverage marketing to African Americans. American journal of public health. 2008 Sep;98(9):1616-29. | Design - review |
| Harris JL, Graff SK. Protecting children from harmful food marketing: options for local government to make a difference. Preventing chronic disease. 2011 Sep;8(5). | Design – opinion paper |
| Hawkesworth S, Silverwood RJ, Armstrong B, Pliakas T, Nanchahal K, Sartini C, Amuzu A, Wannamethee G, Atkins J, Ramsay SE, Casas JP. Investigating the importance of the local food environment for fruit and vegetable intake in older men and women in 20 UK towns: a cross-sectional analysis of two national cohorts using novel methods. International Journal of Behavioral Nutrition and Physical Activity. 2017 Dec;14(1):1-4. | Intervention – not food and drink marketing |
| Holston D, Verbois C, Walker B, Hofer R, Wolford B. P102 A Follow-Up Outcome Evaluation of a Comprehensive SNAP-Ed Social Marketing Campaign in Louisiana. Journal of Nutrition Education and Behavior. 2019 Jul 1;51(7):S78-9. | Intervention – not food and drink marketing |
| Hooper R. The first advertising campaign for non-human primates. New Scientist. 2011 June 27. Available from: https://www.newscientist.com/article/dn20618-the-first-advertising-campaign-for-non-human-primates/ | Design – opinion paper |
| Kapetanaki AB, Wills WJ, Danesi G, Spencer NH. Socioeconomic Differences and the Potential Role of Tribes in Young People’s Food and Drink Purchasing Outside School at Lunchtime. International journal of environmental research and public health. 2019 Jan;16(14):2447. | Intervention – outdoor not isolated |
| Knopf, A. In case you haven’t heard…. Alcoholism & Drug Abuse Weekly. 2014 March 17;26(11):8 | Design – opinion paper |
| Kraak V, Englund T, Misyak S, Serrano E. Progress evaluation for the restaurant industry assessed by a voluntary marketing-mix and choice-architecture framework that offers strategies to nudge American customers toward healthy food environments, 2006–2017. International journal of environmental research and public health. 2017 Jul;14(7):760. | Design - review |
| Larson N, Davey CS, Coombes B, Caspi C, Kubik MY, Nanney MS. Food and beverage promotions in Minnesota secondary schools: secular changes, correlates, and associations with adolescents' dietary behaviors. Journal of school health. 2014 Dec;84(12):777-85. | Intervention – outdoor not isolated |
| Lee RE, Heinrich KM, Reese-Smith JY, Regan GR, Adamus-Leach HJ. Obesogenic and youth oriented restaurant marketing in public housing neighborhoods. American journal of health behavior. 2014 Mar 1;38(2):218-24. | Intervention – outdoor not isolated |
| Mah CL, Luongo G, Hasdell R, Taylor NG, Lo BK. A systematic review of the effect of retail food environment interventions on diet and health with a focus on the enabling role of public policies. Current nutrition reports. 2019 Dec;8(4):411-28. | Design - Review |
| Oropeza S, Sadile MG, Phung CN, Cabiles M, Spackman S, Abuan M, Seligman F, Araneta MR. STRIVE, San Diego! Methodology of a community-based participatory intervention to enhance healthy dining at Asian and Pacific Islander restaurants. Journal of nutrition education and behavior. 2018 Mar 1;50(3):297-306. | Design – study protocol (no data) |
| Paquet C, de Montigny L, Labban A, Buckeridge D, Ma Y, Arora N, Dubé L. The moderating role of food cue sensitivity in the behavioral response of children to their neighborhood food environment: a cross-sectional study. International Journal of Behavioral Nutrition and Physical Activity. 2017 Dec;14(1):1-2. | Intervention – outdoor not isolated |
| Physicians committee. ‘Beans not beef’. Good Medicine. 2021 Summer; 3:p.8 | Design – opinion paper |
| Prowse, R. Food marketing to children in Canada: a settings-based scoping review on exposure, power and impact. Health promotion and chronic disease prevention in Canada: research, policy and practice. 2017 Sep;37(9):274. | Design - review |
| Reger B, Wootan MG, Booth-Butterfield S. A comparison of different approaches to promote community-wide dietary change. American journal of preventive medicine. 2000 May 1;18(4):271-5. | Intervention – not food or drink marketing |
| Reger-Nash B, Wootan MG, Booth-Butterfield S, Cooper L. PEER REVIEWED: The Cost-Effectiveness of 1% Or Less Media Campaigns Promoting Low-Fat Milk Consumption. Preventing Chronic Disease. 2005 Oct;2(4). | Intervention – not food or drink marketing |
| Robles B, Blitstein JL, Lieberman AJ, Barragan NC, Gase LN, Kuo T. The relationship between amount of soda consumed and intention to reduce soda consumption among adults exposed to the Choose Health LA ‘Sugar Pack’health marketing campaign. Public health nutrition. 2015 Oct;18(14):2582-91. | Intervention – not food or drink marketing |
| Saavedra-Garcia L, Meza-Hernández M, Yabiku-Soto K, Hernández-Vásquez A, Kesar HV, Mejia-Victorio C, Diez-Canseco F. Food and beverage supply and advertising in schools and their surroundings in Metropolitan Lima. An exploratory study. Revista Peruana de Medicina Experimental y Salud Pública. 2021 Feb 3;37:726-32. | Intervention - not outdoor (advertising in schools) |
| Schwartz MB, Schneider GE, Choi YY, Li X, Harris J, Andreyeva T, Hyary M, Vernick NH, Appel LJ. Association of a community campaign for better beverage choices with beverage purchases from supermarkets. JAMA internal medicine. 2017 May 1;177(5):666-74. | Intervention – not food or drink marketing |
| Scully M, Wakefield M, Niven P, Chapman K, Crawford D, Pratt IS, Baur LA, Flood V, Morley B, NaSSDA Study Team. Association between food marketing exposure and adolescents’ food choices and eating behaviors. Appetite. 2012 Feb 1;58(1):1-5. | Intervention – outdoor not isolated |
| Signal LN, Jenkin GL, Barr MB, Smith M, Chambers TJ, Hoek J, Mhurchu CN. Prime minister for a day: children's views on junk food marketing and what to do about it. NZ Med J. 2019 Mar 29;132(1492):36-45. | Design - qualitative |
| Signal LN, Smith MB, Barr M, Stanley J, Chambers TJ, Zhou J, Duane A, Jenkin GL, Pearson AL, Gurrin C, Smeaton AF. Kids’ Cam: an objective methodology to study the world in which children live. American Journal of Preventive Medicine. 2017 Sep 1;53(3):e89-95. | Design – methodology paper |
| Tarabashkina L, Quester P, Crouch R. Food advertising, children’s food choices and obesity: Interplay of cognitive defences and product evaluation: An experimental study. International Journal of Obesity. 2016 Apr;40(4):581-6. | Intervention – no outdoor marketing |
| Truman E, Elliott C. Identifying food marketing to teenagers: a scoping review. International Journal of Behavioral Nutrition and Physical Activity. 2019 Dec;16(1):1-0. | Design - review |
| Velazquez CE, Black JL, Potvin Kent M. Food and beverage marketing in schools: a review of the evidence. International journal of environmental research and public health. 2017 Sep;14(9):1054. | Design – review |
| Volpe RJ. Promotional competition between supermarket chains. Review of Industrial Organization. 2013 Feb 1;42(1):45-61. | Intervention – no outdoor marketing |
| Walton M, Waiti J, Signal L, Thomson G. Identifying barriers to promoting healthy nutrition in New Zealand primary schools. Health Education Journal. 2010 Mar;69(1):84-94. | Design – qualitative |
| Wechsler H, Wernick SM. A social marketing campaign to promote low-fat milk consumption in an inner-city Latino community. Public Health Reports. 1992 Mar;107(2):202. | Intervention – not food and drink marketing |

Grey

| Link | Reason for exclusion |
| --- | --- |
| Achieng, JA  Effectiveness of billboard advertising; a case of soft drinks in Nairobi | Intervention - Recall |
| Barr, ML.  https://ourarchive.otago.ac.nz/handle/10523/8097 | Duplicate |
| Christiansen, E  https://mospace.umsystem.edu/xmlui/handle/10355/57614 | Design - qualitative |
| Egli, VC  https://openrepository.aut.ac.nz/handle/10292/12370 | Intervention – methodology development |
| Elsen, M  https://research.tilburguniversity.edu/en/publications/thin-and-thicker-slices-how-advertising-effectiveness-depends-on- | Intervention - not food and drink marketing |
| Fomina, N  https://www.theseus.fi/handle/10024/127929 | Intervention – Recognition |
| Sustain: https://www.sustainweb.org/publications/taking_down_junk_food_ads/ | Design - qualitative |
| Wray, A.  https://ir.lib.uwo.ca/etd/7272/ | Embargoed (not available) |
